# Single-photon smFRET. III. Application to pulsed illumination

**DOI:** 10.1016/j.bpr.2022.100088

**Published:** 2022-11-25

**Authors:** Matthew Safar, Ayush Saurabh, Bidyut Sarkar, Mohamadreza Fazel, Kunihiko Ishii, Tahei Tahara, Ioannis Sgouralis, Steve Pressé

**Affiliations:** 1 Center for Biological Physics, Arizona State University, Tempe, Arizona; 2 Department of Mathematics and Statistical Science, Arizona State University, Tempe, Arizona; 3 Department of Physics, Arizona State University, Tempe, Arizona; 4 Molecular Spectroscopy Laboratory, RIKEN, 2-1 Hirosawa, Wako, Saitama, Japan; 5 Ultrafast Spectroscopy Research Team, RIKEN Center for Advanced Photonics (RAP), 2-1 Hirosawa, Wako, Saitama, Japan; 6 Department of Mathematics, University of Tennessee Knoxville, Knoxville, Tennessee; 7 School of Molecular Sciences, Arizona State University, Phoenix, Arizona

## Abstract

Förster resonance energy transfer (FRET) using pulsed illumination has been pivotal in leveraging lifetime information in FRET analysis. However, there remain major challenges in quantitative single-photon, single-molecule FRET (smFRET) data analysis under pulsed illumination including 1) simultaneously deducing kinetics and number of system states; 2) providing uncertainties over estimates, particularly uncertainty over the number of system states; and 3) taking into account detector noise sources such as cross talk and the instrument response function contributing to uncertainty; in addition to 4) other experimental noise sources such as background. Here, we implement the Bayesian nonparametric framework described in the first companion article that addresses all aforementioned issues in smFRET data analysis specialized for the case of pulsed illumination. Furthermore, we apply our method to both synthetic as well as experimental data acquired using Holliday junctions.

## Why it matters?

In the first companion article of this series, we developed new methods to analyze noisy single-molecule Förster resonance energy transfer data. These methods eliminate the requirement of a priori specifying the dimensionality of the physical model describing a molecular complex’s kinetics. Here, we apply these methods to experimentally obtained datasets with samples illuminated by laser pulses at regular time intervals. In particular, we study conformational dynamics of Holliday junctions.

## Introduction

Among the many fluorescence methods available (1,2,3,4,5,6,7), single-molecule Förster resonance energy transfer (smFRET) has been useful in probing interactions and conformational changes on nanometer scales (8,9,10,11,12). This is typically achieved by estimating FRET efficiencies (and system states) at all instants of an smFRET trace and subsequently estimating transition rates. Furthermore, among different FRET modalities, FRET efficiencies are most accurately determined under pulsed illumination (13,14,15), where the FRET dyes are illuminated by short laser bursts at known times.

Under this illumination procedure, photon arrival times are recorded with respect to the immediately preceding pulse, thereby facilitating an accurate estimation of fluorescence lifetimes as well as FRET rates. As such, in this article, we will focus on single-photon smFRET analysis under pulsed illumination.

Under pulsed illumination, information on kinetic parameters present in smFRET data is traditionally learned by binned photon methods, thereby eliminating lifetime information altogether (16,17,18); bulk correlative methods (19,20,21); and single-photon methods (14,22,23). However, these methods are parametric, i.e., require fixing the number of system states a priori, and necessarily only learn system kinetics even though information on the number of system states is encoded in the data.

In this article, we implement a general smFRET analysis framework that was presented in Sec. 2.5.1 of the first companion manuscript (24) for the case of pulsed illumination to learn full distributions. In other words, probability distributions over parameters take into account uncertainties from all existing sources such as cross talk and background. These parameters include the system transition probabilities and photophysical rates, that is, donor and acceptor relaxation and FRET rates, with special attention paid to uncertainty arising from sources such as inherent stochasticity in photon arrival times and detectors. As our main concern is deducing the number of system states using single-photon arrivals while incorporating detector effects, we leverage the formalism of infinite hidden Markov models (iHMMs) (25,26,27,28,29,30) within the Bayesian nonparametric (BNP) paradigm (25,26,31,32,33,34,35,36,37,38). The iHMM framework assumes an a priori infinite number of system states with associated transition probabilities, where the number of system states warranted by input data is enumerated by those states most visited over the course of the system state trajectory.

Next, to benchmark our BNP-FRET sampler, we analyzed synthetic and experimental smFRET data acquired using a single confocal microscope with pulsed illumination optimized to excite donor dyes.

In particular, we employ a broad range of experimental data acquired from Holliday junctions (HJs) with an array of different kinetic rates due to varying buffer concentration of $MgCl_{2}$ (39,40,41,42).

## Materials and methods

### Terminology convention

To be consistent throughout our three-part article, we precisely define some terms as follows.

1. A macromolecular complex under study is always referred to as a system.
2. The configurations through which a system transitions are termed system states, typically labeled using σ.
3. FRET dyes undergo quantum mechanical transitions between photophysical states, typically labeled using ψ.
4. A system-FRET combination is always referred to as a composite.
5. A composite undergoes transitions among its superstates, typically labeled using φ.
6. All transition rates are typically labeled using λ.
7. The symbol N is generally used to represent the total number of discretized time windows, typically labeled with n.
8. The symbol $w_{n}$ is generally used to represent the observations in the n-th time window.

### Forward model and inverse strategy

In this section, we first briefly illustrate the adaptation of the general formalism described in our first companion article (24) to the pulsed illumination case. Next, we present a specialized inference procedure. The details of the framework not provided herein can be found in the supporting material.

As before, we consider a molecular complex labeled with a donor-acceptor FRET pair. As the molecular complex transitions through its $M_{\sigma }$ system states indexed by $\sigma_{1:M_{\sigma }}$, laser pulses (optimized to excite the donor) separated by time τ may excite either the donor or acceptor to drive transitions among the photophysical states, $\psi_{1:M_{\psi }}$, as defined in the first companion article (24). Such photophysical transitions lead to photon emissions that may be detected in either donor or acceptor channels. The set of N observations, e.g., photon arrival times, from N pulses are recorded as

(1) $$w=\left\{w_{1},w_{2},\ldots ,w_{N}\right\}\text{.}$$

Here, each individual measurement is a pair $w_{n}=\left(\mu_{n}^{d},\mu_{n}^{a}\right)$, where $\mu_{n}^{d}$ and $\mu_{n}^{a}$ are the recorded arrival times (also known as microtimes) after the n-th pulse in both donor and acceptor channels, respectively. In cases where there is no photon detection, we denote the absent microtimes with $\mu_{n}^{d}=\emptyset$ and $\mu_{n}^{a}=\emptyset$ for donor and acceptor channels, respectively.

**Figure 1:**
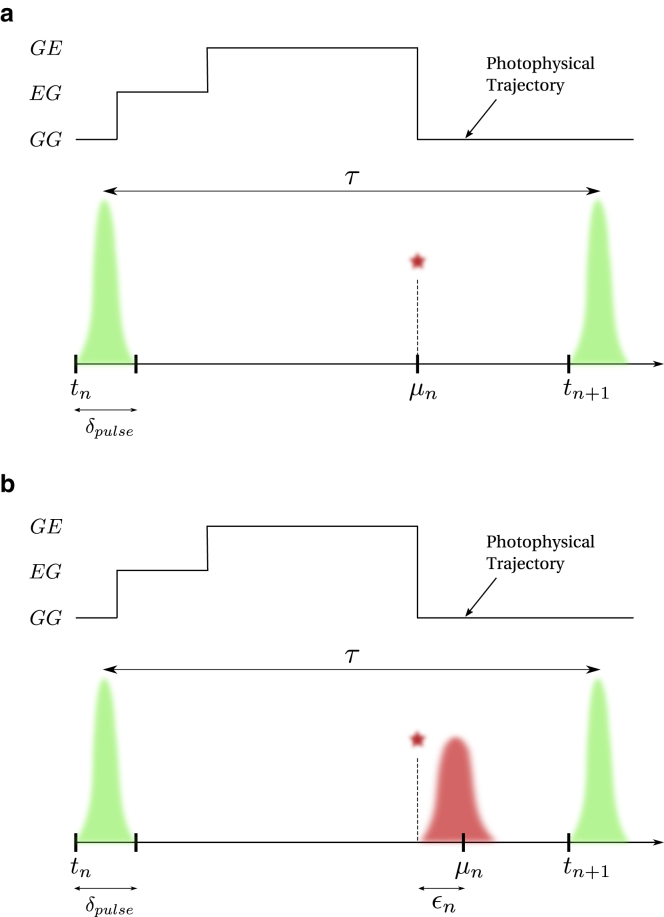
Events over a pulsed illumination experiment pulse window. Here, the beginning of the n-th interpulse window of size τ is marked by time $t_{n}$. The FRET labels originally in state GG (donor and acceptor, respectively, in ground states) are excited by a high intensity burst (shown in *green*) to the state EG (only donor excited) for a very short time, $\delta_{pulse}$. If FRET occurs, the donor transfers its energy to the acceptor and resides in the ground state, leaving the FRET labels in the GE state (only acceptor excited). The acceptor then emits a photon to be registered by the detector at microtime $\mu_{n}$. When using ideal detectors, the microtime is the same as the photon emission time as shown in (*a*). However, when the timing hardware has jitter (shown in *red*), a small delay $\varepsilon_{n}$ is added to the microtime as shown in (*b*). For convenience, we have reproduced this figure from our first companion article (24).

As is clear from Fig. 1, smFRET traces are inherently stochastic due to the nature of photon excitation, emission, and noise introduced by detector electronics. To analyze such stochastic systems, we begin with the most generic likelihood derived in Eq. 51 of the first companion article (24),

(2) $$L\propto \rho_{start}Q_{1}\ldots Q_{n}\ldots Q_{N}\rho_{norm}^{T}\text{,}$$

where $\rho_{start}$ is the initial probability vector for the system-FRET composite to be in one of $M_{\phi }=\left(M_{\sigma }\times M_{\psi }\right)$ superstates, and $\rho_{norm}$ is a vector that sums the elements of the propagated probability vector. Here, we recall that $Q_{n}$ is the transition probability matrix between pulses at $t_{n}$ and $t_{n+1}$, characterizing system-FRET composite transitions among superstates.

The propagators $Q_{n}$ above adopt different forms depending on whether a photon is detected or not during the associated period. Their most general forms are derived in the section on illumination features in the first companion article (24). However, these propagators involve computationally expensive integrals, and thus we make a few approximations here as follows: 1) we assume that the system state remains the same over an interpulse period since typical system kinetic timescales (typically 1 ms or more) are much longer than interpulse periods (≈100 ns) (41,43), and 2) the interpulse period (≈100 ns) is longer than the donor and acceptor lifetimes (≈ a few ns) (41,43) such that they relax to the ground state before the next pulse. Furthermore, we will demonstrate a specialized sampling scheme under these physically motivated approximations.

The immediate implications of the first assumption are that the system transitions may now, to a good approximation, only occur at the beginning of each pulse. Consequently, the evolution of the FRET pair between two consecutive pulses is now exclusively photophysical, as the system state remains the same during interpulse times. As such, the system now evolves in equally spaced discrete time steps of size τ, where the system state trajectory can be written as

$$s_{1:N}=\left\{s_{1},s_{2},\ldots ,s_{n},\ldots ,s_{N-1},s_{N}\right\}\text{,}$$

where $s_{n}$ is the system state between pulses n and n+1. The stochastic evolution of the system states in such discrete steps is then determined by the transition probability matrix designated by $\Pi_{\sigma }$. For example, in the simplest case of a molecular complex with two system states $\sigma_{1:2}$, this matrix is computed as follows:

(3) $$\Pi_{\sigma }=\exp\left(\tau \left[\begin{array}{cc} {}* & \lambda_{\sigma_{1}\to \sigma_{2}}\\ \lambda_{\sigma_{2}\to \sigma_{2}} & * \end{array}\right]\right)=\left[\begin{array}{cc} \pi_{\sigma_{1}\to \sigma_{1}} & \pi_{\sigma_{1}\to \sigma_{2}}\\ \pi_{\sigma_{2}\to \sigma_{1}} & \pi_{\sigma_{2}\to \sigma_{2}} \end{array}\right]\text{,}$$

where the matrix in the exponential contains transition rates among the system states and the ∗ represents the negative row sum.

Next, by assumption two, we can further suppose that the fluorophores always start in the ground state at the beginning of every pulse. As a result, we treat pulses independently and write the probability of observation $w_{n}$ as

(4) $$p\left(w_{n}|s_{n},G_{\psi }\right)=\rho_{ground}Q_{n}^{\psi }\left(s_{n}\right)\rho_{norm}^{T}\text{,}$$

where $\rho_{ground}$ denotes the probability vector when the FRET pair is in the ground state at the beginning of each pulse, $G_{\psi }$ is the generator matrix with only photophysical transition rates, and $Q_{n}^{\psi }\left(s_{n}\right)$ is the photophysical propagator for the n-th interpulse period.

We further organize the observation probabilities of Eq. 4 into a newly defined detection matrix $D_{n}^{\sigma }$ with its elements given by ${\left(D_{n}^{\sigma }\right)}_{s_{n}\to \sigma_{j}}=p\left(w_{n}|s_{n},G_{\psi }\right)$. Here, we note that the index j does not appear on the right-hand side because the system state does not change during an interpulse window, resulting in the independence of observation probability from the next system state, $s_{n+1}$. The explicit formulas for the observation probabilities are provided in the supporting material.

Now, using the matrix $D_{n}^{\sigma }$, we define the reduced propagators for each interpulse period as

(5) $$\Pi_{n}^{\sigma }=\Pi_{\sigma }⊙D_{n}^{\sigma }\text{,}$$

where ⊙ denotes the element-by-element product.

Finally, using these simplified propagators, we can write the likelihood for an smFRET trace under pulsed illumination as

(6) $$L=p\left(w|\rho_{start},\Pi_{\sigma },G_{\psi }\right)\propto \rho_{start}\Pi_{1}^{\sigma }\Pi_{2}^{\sigma }\ldots \Pi_{N}^{\sigma }\rho_{norm}^{T}\text{,}$$

as also introduced in the section on illumination features in the first companion article (24). This form of the likelihood is advantageous in that it allows empty pulses to be computed as a simple product, greatly reducing computational cost.

In the following, we first illustrate a parametric inference procedure assuming a given number of system states. We next generalize the procedure developed to the nonparametric case to deduce the number of system states along with the rest of parameters.

#### Inference procedure: Parametric sampler

With the likelihood at hand, we construct the posterior as follows

(7) $$p\left(\rho_{start},\Pi_{\sigma },G_{\psi }|w\right)\propto p\left(w|\rho_{start},\Pi_{\sigma },G_{\psi }\right)p\left(\rho_{start}\right)p\left(G_{\psi }\right)p\left(\Pi_{\sigma }\right)\text{,}$$

where we assume that the unknown parameters, including the initial probability vector, $\rho_{start}$, the photophysical transition rates in the generator matrix $G_{\psi }$, and the transition probabilities among system states in propagator $\Pi_{\sigma }$ are independent, allowing us to conveniently write the prior on these parameters as a product (the last three terms on right hand side). Here, we can sample the set of unknowns using the above posterior with the Gibbs sampling procedure described in the first companion article (see the section describing inverse strategy in (24)). However, a computationally more convenient inference procedure that allows direct sampling is accomplished by writing the posterior of Eq. 7 as a marginalization (sum) over state trajectories as follows

(8) $$\begin{array}{cc} p\left(\rho_{start},\Pi_{\sigma },G_{\psi }|w\right) & =\sum_{s_{1:N}}p\left(\rho_{start},\Pi_{\sigma },G_{\psi },s_{1:N}|w\right)\\ & \propto \sum_{s_{1:N}}p\left(w|\Pi_{\sigma },G_{\psi },s_{1:N}\right)p\left(\rho_{start}\right)p\left(G_{\psi }\right)p\left(\Pi_{\sigma }\right)p\left(s_{1:N}|\rho_{start},\Pi_{\sigma }\right)\text{,} \end{array}$$

where $s_{1:N}=\left\{s_{1},s_{2}\ldots ,s_{N}\right\}$ denotes a system state trajectory. Now, we can use the nonmarginal posterior

(9) $$p\left(\rho_{start},\Pi_{\sigma },G_{\psi },s_{1:N}|w\right)\propto p\left(w|\Pi_{\sigma },G_{\psi },s_{1:N}\right)p\left(\rho_{start}\right)p\left(G_{\psi }\right)p\left(\Pi_{\sigma }\right)p\left(s_{1:N}|\rho_{start},\Pi_{\sigma }\right)$$

to sample the trajectory $s_{1:N}$, which, in turn, allows direct sampling of the elements of propagator $\Pi_{\sigma }$ described shortly. For priors on $\rho_{start}$ and rates in $G_{\psi }$, we, respectively, use Dirichlet and Gamma distributions similar to Eqs. 65 and 66 of the first companion article (24). We sample the system state trajectory $s_{1:N}$ by recursively sampling the states using a forward filtering backward sampling algorithm described in section S4.3.

Finally, for each row in the propagator $\Pi_{\sigma }$, we use a Dirichlet prior

(10) $$\pi_{m}∼Dirichlet\left(\alpha \beta \right),m=1,2,\ldots ,M_{\sigma }\text{,}$$

where $M_{\sigma }$ is the number of system states and $\pi_{m}$ denotes the m-th row of the propagator. Here, the hyperparameters α and β are, respectively, the concentration parameter and a vector of length $M_{\sigma }$ described in the first companion article (see Section 3.2.2 of (24)). We can now directly generate samples for the transition probability vectors $\pi_{m}$ of length $M_{\sigma }$ via prior-likelihood conjugacy as (see section S4.3)

$$\pi_{m}∼Dirichlet\left(n_{m}+\alpha \beta \right),m=1,2,\ldots ,M_{\sigma }^{\max}\text{,}$$

where the vector $n_{m}$ collects the number of times each transition out of system state $\sigma_{m}$ occurs obtained using the system state trajectory.

After constructing the posterior, we can make inferences on the parameters by drawing samples from the posterior. However, as the resulting posterior has a nonanalytical form, it cannot be directly sampled. Therefore, we develop a Markov chain Monte Carlo sampling procedure (37,38,44,45,46,47) to draw samples from the posterior.

Our Markov chain Monte Carlo sampling scheme follows a Gibbs sampling technique, sweeping through updates of the set of parameters in the following order: 1) photophysical transition rates including donor relaxation rates $\lambda_{d}$ (inverse of donor lifetime), acceptor relaxation rate $\lambda_{a}$ (inverse of acceptor lifetime), FRET rates $\lambda_{\sigma_{1:M_{\sigma }}}^{FRET}$ for each system state, and excitation rate (inverse of excitation probability $\pi_{ex}$) using the Metropolis-Hastings(MH) step; 2) transition probabilities between system states, $\pi_{1:M_{\sigma }}$, by directly drawing samples from the posterior; 3) the system states trajectory, S, using a forward-backward sampling procedure (48); and 4) the initial probabilities, $\rho_{start}$, by taking direct samples. In the end, the chains of samples drawn can be used for subsequent numerical analysis.

#### Inference procedure: Nonparametrics sampler

The smFRET data analysis method illustrated above assumes a given number of system states, $M_{\sigma }$. However, in many applications, the number of system states is not specified a priori. Here, we describe a generalization of our parametric method to address this shortcoming and estimate the number of system states simultaneously along with other unknown parameters.

We accomplish this by modifying our previously introduced parametric posterior as follows. First, we suppose an infinite number of system states $\left(M_{\sigma }\to \infty \right)$ for the likelihood introduced previously and learn the transition matrix $\Pi_{\sigma }$. The number of system states can then be interpreted as those appreciably visited over the course of the trajectory.

To incorporate this infinite system state space into our inference strategy, we leverage the iHMMs (25,26,28,29,30) from the BNP repertoire, placing a hierarchical Dirichlet process prior over the infinite set of system states as described in the first companion article (the inverse strategy section in (24)). However, as detailed in the first companion manuscript (the inverse strategy section in (24)), dealing with an infinite number of random variables, though feasible, is not computationally efficient, and we approximate this infinite value with a large number, $M_{\sigma }^{\max}$, reducing our hierarchical Dirichlet process prior to

$$\begin{array}{cc} \beta & ∼Dirichlet\left(\frac{\gamma }{M_{\sigma }^{\max}},\ldots ,\frac{\gamma }{M_{\sigma }^{\max}}\right)\text{,}\\ \pi_{m} & ∼Dirichlet\left(\alpha \beta \right)\;,m=1,\ldots ,M_{\sigma }^{\max}\text{.} \end{array}$$

Here, β denotes the base probability vector of length $M_{\sigma }^{\max}$ serving itself as a prior on the probability transition matrix $\Pi_{\sigma }$, and $\pi_{m}$ is the m-th row of $\Pi_{\sigma }$. Moreover, γ is a positive scalar hyperparameter of the Dirichlet process prior often chosen to be one. As such, we ascribe identical weights across the state space a priori for computational convenience (28,29,49).

Now, equipped with the nonparametric posterior, we proceed to simultaneously make inferences on transition probabilities, excited-state escape rates, and the remaining parameters. To do so, we employ the Gibbs sampling scheme detailed in the inverse strategy section in the first companion article (24), except that we must now also sample the system state trajectory $s_{1:N}$. More details on the overall sampling scheme are found in section S4 of the supporting material.

## Results

The main objective of our method is to learn full distributions over 1) transition probabilities among $M_{\sigma }^{\max}$ system states determining, in turn, the corresponding system transition rates and the effective number of system states, and 2) photophysical transition rates, including FRET rates $\lambda_{1:M}^{FRET}$, and fluorophores’ relaxation rates (inverse of lifetimes) $\lambda_{a}$ and $\lambda_{d}$.

To sample from distributions over these parameters, the BNP-FRET sampler requires input data comprised of photon arrival time traces from both donor and acceptor channels as well as a set of precalibrated input parameters including camera effects such as cross talk matrix and detection efficiency (see Sec. 2.4 and example V of the first companion article (24)); background emission (see the section on background in the first companion article and Section S2.4); and the instrument response function (IRF) (see illumination features sectoin in the first companion article (24) and Section S2.3).

Here, we first show that our method samples posteriors over a set of parameters employing realistic synthetic data generated using the Gillespie algorithm (50) to simulate system and photophysical transitions while incorporating detector artefacts such as crosstalk (see the synthetic data generation section in the first companion article (24)). The list of parameters used in data generation for all figures is provided in Section S6. Furthermore, prior hyperparameters used in the analysis of synthetic and experimental data are listed in Section S3.

**Figure 2:**
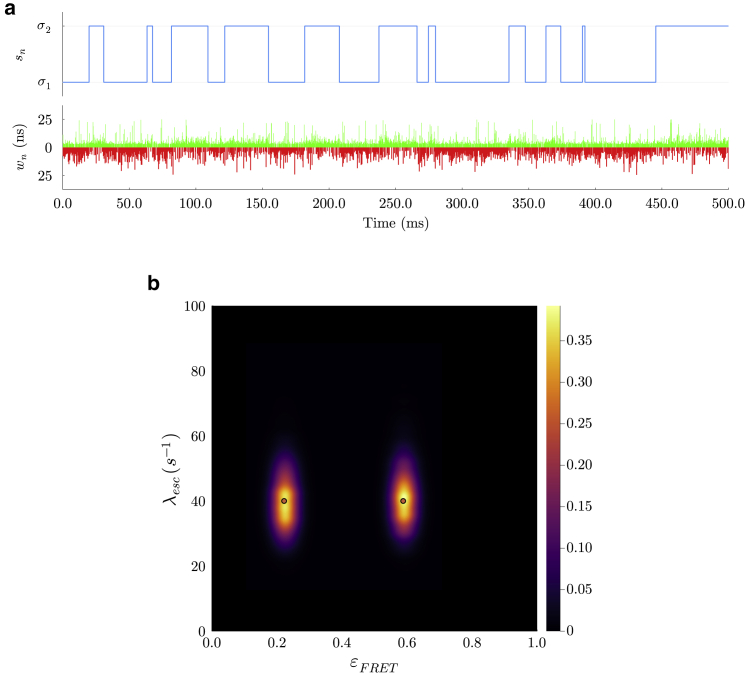
Analysis on synthetic data for a system with two system states. In (*a*), we show a section of synthetic data produced with the values in Table S2. Furthermore, the system state trajectory is shown in blue. Below this, the arrival times of donor and acceptor photons $\mu_{n}^{d}$ and $\mu_{n}^{a}$ are shown in green and red, respectively. In (*b*), we plot the bivariate distribution over escape rates and FRET efficiencies. The ground truth is shown with red dots corresponding to an escape rate of 40 s^−1^ and FRET efficiencies of 0.22 and 0.59. $\lambda_{esc}$$\varepsilon_{FRET}$. As seen, the BNP-FRET sampler clearly distinguishes two system states with maximum a posteriori (MAP) estimates for the associated escape rates of $\approx 38_{-7}^{+7}$ and $\approx 40_{-7}^{+7}$ s^−1^ and for FRET efficiencies of $\approx 0.21_{-0.03}^{+0.03}$ and $\approx 0.59_{-0.03}^{+0.03}$. We have smoothed the distributions using kernel density estimation for illustration purposes only.

**Figure 3:**
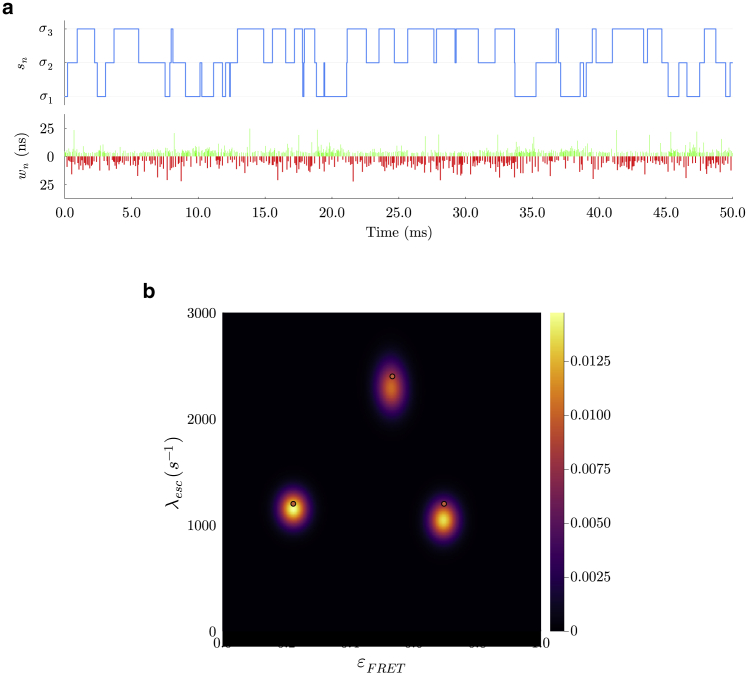
Analysis on synthetic data for three system states. In (*a*), we have a section of synthetic data produced with the values from Table S3. The system state trajectory is seen in blue. Below this, the arrival times of donor and acceptor photons $\mu_{n}^{d}$ and $\mu_{n}^{a}$ are shown in green and red, respectively. In (*b*), we plot the distribution over escape rates and FRET efficiencies $\varepsilon_{FRET}$. The red dots show ground truths corresponding to escape rates of 1,200, 2,400, and 1,200 s^−1^ and FRET efficiencies of 0.22, 0.53, and 0.7. From our maximum a posteriori (MAP) estimate, $\lambda_{esc}$ $\varepsilon_{FRET}$ we clearly see three system states with escape rates of ${1,100}_{-60}^{+60}$, ${2,300}_{-128}^{+131}$, and ${1,050}_{-80}^{+80}$ s^−1^.

We first show that our method works for the simplest case of slow transitions compared with the interpulse period (25 ns) with two system states using synthetic data (see Fig. 2). Next, we proceed to tackle more challenging synthetic data with three system states and higher transition rates (Fig. 3). We show that our nonparametric algorithm correctly infers system transition probabilities and thus the number of system states (see Fig. 3).

**Figure 4:**
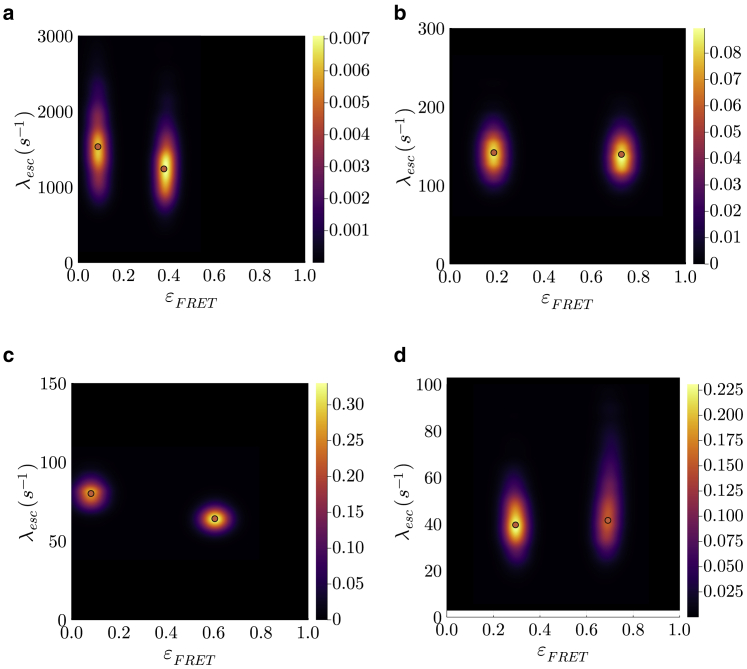
The bivariate posterior for the conformational transition rates $\lambda_{esc}$ and FRET efficiencies $\varepsilon_{FRET}$ for experimental data acquired in the presence of different HJ concentrations. Here, we show our bivariate posteriors where red dots show MAP estimates. In (*a*), we show the posterior for a sample with 1 mM$MgCl_{2}$. We report escape rates of ${1,530}_{-550}^{+500}$ and ${1,240}_{-420}^{+420}$ s^−1^ in this case. The posterior for a sample with 3 mM$MgCl_{2}$ is shown in (*b*). We report escape rates of $140_{-38}^{+38}$ and $142_{-32}^{+32}$ s^−1^ for this case. In (*c*), we show our posterior for a sample with 5 mM$MgCl_{2}$. Here, we report escape rates of $64_{-9}^{+9}$ and $80_{-10}^{+10}$ s^−1^. The posterior in (*d*) is for a sample with 10 mM$MgCl_{2}$. We report escape rates of $39_{-12}^{+17}$ and $41_{-12}^{+23}$ s^−1^.

After demonstrating the performance of our method using synthetic data, we use experimental data to investigate the kinetics of HJs under different $MgCl_{2}$ concentrations in buffer (see Fig. 4).

### Simulated data analysis

To help validate BNPs on smFRET single-photon data, we start with a simple case of a two-state system and select kinetics similar to those of the experimental data sets, c.f., the HJ in 10 mm $MgCl_{2}$, with escape rates of 40 s^−1^ for both system states (51). The generated system state trajectory and photon traces over a period of 500 ms from both channels are shown in Fig. 2 *a*.

Fig. 2*b* shows the bivariate posterior distribution over FRET efficiencies, $\varepsilon_{FRET}$, defined as $\varepsilon_{FRET}=\lambda_{FRET}/\left(\lambda_{FRET}+\lambda_{d}\right)$, and system escape rates, i.e., obtained by computing the logarithm of the propagator matrix, with two peaks corresponding to the two system states most visited by the sampler. Furthermore, the ground truths, designated by red dots, fall within the posterior with a relative error of less than 3% from the posterior modes. The results for the remaining parameters, including donor and acceptor transition rates, FRET transition rates, and system transition probabilities, are presented in Section S7.

To showcase the critical role played by BNPs, we also consider the more difficult case of a sample with three system states and faster system state kinetics ranging over 1,200–2,500 $s^{-1}$. We do so by simulating photon traces in both donor and acceptor channels over a period of ∼150 ms. A 50 ms section of the synthetic photon trace is shown in Fig 3 *a*.

Using direct photon arrivals from the generated photon trace, we find that the most probable system state trajectories sampled by BNP-FRET visit the correct number of system states, as shown in Fig 3 *b*, while inferring all other parameters. Furthermore, the BNP-FRET sampler estimates the system transition rates and thus the escape rates (i.e., sum of transition rates out of a given state) where the ground-truth escape rates differ from the posterior peaks by a relative average error of less than 8%. The results for the remaining parameters are provided in Section S7.

### Experimental data analysis: HJ

In this section, we benchmark our method over a wide range of kinetic rates employing experimental data acquired using HJ under varying buffer $MgCl_{2}$ concentrations (15,51).

HJs are four-way double-helical DNA junctions existing in various structural configurations (41,52,53). When not interacting with multivalent metal ions, electrostatic repulsion between negatively charged phosphate groups of the four helical arms forces HJs to assume a wide configuration where the arms lie along the two diagonals of a square. However, in the presence of ions, such as Mg^2+^, interaction with the phosphate groups results in electrostatic screening. This reduced repulsion induces transitions to what is believed to be primarily two compact stacked configurations/conformations. The transitions between both conformations necessitates passing through the intermediate open configuration. Since, at high ion concentrations, displacing ions away from the phosphate group becomes increasingly difficult, in this scenario, we anticipate smaller transition rates between both conformations.

The HJ kinetic rates have been studied using both fluorescence lifetime correlation spectroscopy (15) and HMM analysis (54) on diffusing HJs assuming a priori a pair of high and low FRET system states. As expected, these previous studies show kinetic rates decreasing with increasing $MgCl_{2}$ concentrations (41,43) and correspondingly longer dwells.

Here, our method, free from averaging and binning that are otherwise common in HMM analysis, is particularly well suited to learn the rapid kinetics at low Mg^2+^ concentrations. We apply our BNP-FRET to data acquired from HJs at 1, 3, 5, and 10 mM $MgCl_{2}$ concentrations and sample the photophysical transition rates and the system transition probabilities.

The acquired bivariate posterior distributions over the FRET efficiencies and escape rates (computed via the logarithm of the system transition probability matrix $\Pi_{\sigma }$) are presented in Fig. 4. Moreover, estimates for the other parameters can be found in Section S7. We note that our results are obtained on a single-molecule basis with a photon budget of $10^{4}–10^{5}$ photons.

For all four concentrations (see Fig. 4), our BNP-FRET sampler most frequently visited only two system states, while this was given as an input to the other analysis methods (15,54). Moreover, both escape rates are found to have similar values with an average of approximately 1,400s^−1^ (1 mM $MgCl_{2}$), 140s^−1^ (3 mM $MgCl_{2}$), 72s^−1^ (5 mM $MgCl_{2}$), and 41 s^−1^ (10 mM $MgCl_{2}$). These escape rates are in close agreement with values reported by fluorescence lifetime correlation spectroscopy and H2MM methods (15,54) of ≈1,300 s^−1^ (1 mM $MgCl_{2}$), ≈170 (3 mM $MgCl_{2}$), ≈100 (5 mM $MgCl_{2}$), and ≈60 s^−1^ (10 mM $MgCl_{2}$), which lie well within the bounds of our posteriors shown in Fig. 4 while simultaneously, and self-consistently, learning a number of system states.

### Experimental data acquisition

In this section, we describe the protocol for preparing the surface-immobilized HJ sample labeled with a FRET pair and the procedure for recording smFRET traces from individual immobilized molecules. The sample preparation and recording of data follow previous work (55).

#### Sample preparation

The HJ used in this work consists of four DNA strands whose sequences are as follows:

R-strand: 5′-CGA TGA GCA CCG CTC GGC TCA ACT GGC AGT CG-3′
H-strand: 5′-CAT CTT AGT AGC AGC GCG AGC GGT GCT CAT CG-3′
X-strand: 5′-biotin-TCTTT CGA CTG CCA GTT GAG CGC TTG CTA GGA GGA GC-3′
B-strand: 5′-GCT CCT CCT AGC AAG CCG CTG CTA CTA AGA TG-3’

For surface immobilization, the X-strand was labeled with biotin at the 5′ end. For FRET measurements, the donor (ATTO-532) and acceptor (ATTO-647N) dyes were introduced into the H- and B-strands, respectively. In both cases, the dyes were labeled to thymine nucleotide at the 6th position from the 5′ ends of the respective strands (shown as T). All DNA samples (labeled or unlabeled) were purchased from JBioS (Shinjuku-ku, Japan) in the high-performance liquid chromatography purified form and were used without any further purification.

The HJ complex was prepared by mixing 1 mM solutions of R-, H-, B-, and X-strands in TN buffer (10 mM Tris-HCl with 50 mm NaCl, pH 8) at a 3:2:3:3 molar ratio, annealing the mixture at 94°C for 4 minutes, and gradually cooling it down (2°C–3°C  min^−1^) to room temperature (25°C ). For smFRET measurements, we used a sample chamber (SecureSeal, GBL621502, Grace Bio-Labs, Bend, OR, USA) with a biotin-PEG-SVA (biotin-poly(ethylene glycol)-succinimidyl valerate)-coated coverslip. The chamber was first incubated with streptavidin (0.1 mg mL^−1^ in TN buffer) for 20 min. This was followed by washing the chamber with TN buffer (3 times) and injection of 1 nM HJ solution (with respect to its H-strand) for 3–10 s. After this incubation period, the chamber was rinsed with TN buffer (3 times) to remove unbound DNA, and it was filled with TN buffer containing 1 mM (or 5 mM) $MgCl_{2}$ and 2 mM Trolox for smFRET measurements.

#### smFRET measurements

The smFRET traces from individual HJs were recorded using a custom-built confocal microscope (Eclipse Ti, Nikon, Tokyo, Japan) equipped with the Perfect Focus System, a sample scanning piezo stage (Nano control B16-055), and a time-correlated single-photon counting module (SPC-130EM, Berlin, Germany).

The broadband light generated by a supercontinuum laser operating at 40 MHz (SC-400-4, Fianium, Southampton, UK) was filtered with a band-pass filter (FF01-525/30, Semrock, West Henrietta, NJ, USA) for exciting the donor dye, ATTO-532. This excitation light was introduced to the microscope using a single-mode optical fiber (P5-460B-PCAPC-1, Thorlabs, Newton, NJ, USA) and directed onto the sample using a dichroic mirror (ZT532/640rpc, Chroma, Cambridge, MA, USA) and a water immersion objective lens (Nikon Plan Apo IR 60×, numerical aperture: 1.27).

The excitation light was focused onto the top surface of the coverslip, and, during measurements, the focusing condition was maintained using the Perfect Focus System. The fluorescence signals were collected by the same objective, passed through the dichroic mirror, and guided to the detection assembly (Thorlabs DFM1/M) using a multimode fiber (Thorlabs M50L02S-A). Note that this multimode fiber (core diameter: 50 *μ*m) also acts as the confocal pinhole. In the detection assembly, the fluorescence signals from the donor and acceptor dyes were separated using a dichroic mirror (ZT633rdc, Chroma Technology, Bellows Falls, VT, USA), filtered using band-pass filters (Chroma ET585/65m for donor and Semrock FF02-685/40 for acceptor), and detected using separate hybrid detectors (Becker and Hickl HPM-100-40-C).

For each detected photon, its macrotime (absolute arrival time from the start of the measurement) was recorded with 25.2 ns resolution and its microtime (relative delay from the excitation pulse) was recorded with 6.1 ps resolution using the time-correlated single-photon counting module operating in time-tagging mode. A router (Becker and Hickl HRT-41) was used to process the signals from the donor and acceptor detectors.

For recording smFRET traces from individual HJs, we first imaged a 10 × 3 *μ*m area of the sample using the piezo stage by scanning it linearly at a speed of 1 *μ*m s^−1^ in the *x* direction and with an increment of 0.1 *μ*m in the *y* direction. Individual HJs appeared as isolated bright spots in the image.

Next, we fitted the obtained donor and acceptor intensity images with multiple 2D Gaussian functions to determine the precise locations of individual HJs. Note that, during this image acquisition, the laser excitation power was kept to a minimum (∼1 *μ*W at the back aperture of the objective lens) to avoid photobleaching the dyes. In addition, we also employed an electronic shutter (Suruga Seiki, Shizuoka, Japan) in the laser excitation path to control the sample excitation as required.

Using the precise locations of individual HJs obtained, we recorded 30 s-long smFRET traces for each molecule by moving them to the center of the excitation beam using the piezo stage. For each trace, the laser excitation was blocked (using the shutter) for the first 5 s and was allowed to excite the sample for the remaining 25 s. Note that the smFRET traces were recorded using 40 *μ*W laser excitation (at the back aperture of the objective lens) to maximize the fluorescence photons emitted from the dyes. We automated the process of acquiring smFRET traces from different molecules sequentially and executed it using a program written in house on Igor Pro (Wavemetrics, Portland, OR, USA).

## Discussion

The sensitivity of smFRET under pulsed illumination has been exploited to investigate many different molecular interactions and geometries (8,9,10,11,56). However, quantitative interpretation of smFRET data faces serious challenges including an unknown number of system states and robust propagation of uncertainty from noise sources such as detectors and background. These challenges ultimately mitigate our ability to determine full distributions over all relevant unknowns and, traditionally, have resulted in data pre- or postprocessing compromising the information otherwise encoded in the rawest form of data: single-photon arrivals.

Here, we provide a general BNP framework for smFRET data analysis starting from single-photon arrivals under a pulsed illumination setting. We simultaneously learn transition probabilities among system states as well as determine photophysical rates by incorporating existing sources of uncertainty such as background and cross talk.

We benchmark our method using both experimental and simulated data. That is, we first show that our method correctly learns parameters for the simplest case with two system states and slow system transition rates. Moreover, we test our method on more challenging cases with more than two states using synthetic data and obtain correct estimations for the system state transition probabilities and thus the number of system states along with the remaining parameters of interest. To further assess our method’s performance, we analyzed experimental data from HJs suspended in solutions with a range of $MgCl_{2}$ concentrations. These data were previously processed using other techniques assuming a fixed number of system states by binning photon arrival times (15).

Despite multiple advantages mentioned above for BNP-FRET, BNPs always come with an added computational cost as they take full advantage of information from single-photon arrival times and all existing sources of uncertainty. For this version of our general BNP method simplified for pulsed illumination, we further reduced the computational complexity by grouping empty pulses together. Therefore, the computational complexity increased only linearly with the number of input photons as the photons are treated independently.

The method described in this paper assumes a Gaussian IRF. However, the developed framework is not limited to a specific form for the IRF and can be used for data collected using any type of IRF by modifying Eq. 4. Furthermore, the framework is flexible in accommodating different illumination techniques such as alternating color pulses, which are typically used to directly excite the acceptor fluorophores. This can be achieved by simple modification of the propagator $Q_{n}^{\psi }$ in Eq. 4. A future extension of this method could relax the assumption of a static sample by adding spatial dependence to the excitation rate as we explored in previous works (35,47,57). This would allow our method to learn the dynamics of diffusing molecules, as well as their photophysical and system state transition rates.

### Code availability

The BNP-FRET software package is available on Github at https://github.com/LabPresse/BNP-FRET.

## Supporting material

Document S1. Supporting material, Figures S1–S7, and Tables S1–S3

Document S2, Article plus supporting material

## References

[1] Weiss S. (1999). Fluorescence spectroscopy of single biomolecules. Science.

[2] Lippincott-Schwartz J., Snapp E., Kenworthy A. (2001). Studying protein dynamics in living cells. Nat. Rev. Mol. Cell Biol..

[3] Huang B., Bates M., Zhuang X. (2009). Super-resolution fluorescence microscopy. Annu. Rev. Biochem..

[4] Lelek M., Gyparaki M.T., Zimmer C. (2021). Single-molecule localization microscopy. Nat. Rev. Methods Primers.

[5] Fazel M., Wester M.J. (2022). Analysis of super-resolution single molecule localization microscopy data: A tutorial. AIP Adv..

[6] Datta R., Heaster T.M., Skala M.C. (2020). Fluorescence lifetime imaging microscopy: fundamentals and advances in instrumentation, analysis, and applications. J. Biomed. Opt..

[7] Garini Y., Young I.T., McNamara G. (2006). Spectral imaging: principles and applications. Cytometry A..

[8] Roy R., Hohng S., Ha T. (2008). A practical guide to single-molecule FRET. Nat. Methods.

[9] Mazal H., Haran G. (2019). Single-molecule FRET methods to study the dynamics of proteins at work. Curr. Opin. Biomed. Eng..

[10] Schuler B. (2013). Single-molecule FRET of protein structure and dynamics - a primer. J. Nanobiotechnol..

[11] Lu M., Ma X., Mothes W. (2019). Associating HIV-1 envelope glycoprotein structures with states on the virus observed by smFRET. Nature.

[12] Mooney S.M., Qiu R., Weninger K.R. (2014). Cancer/testis antigen PAGE4, a regulator of c-Jun transactivation, is phosphorylated by homeodomain-interacting protein kinase 1, a component of the stress-response pathway. Biochemistry.

[13] Wozniak A.K., Schröder G.F., Oesterhelt F. (2008). Single-molecule FRET measures bends and kinks in DNA. Proc. Natl. Acad. Sci. USA.

[14] Chung H.S., Louis J.M., Gopich I.V. (2016). Analysis of fluorescence lifetime and energy transfer efficiency in single-molecule photon trajectories of fast-folding proteins. J. Phys. Chem. B.

[15] Heo W., Hasegawa K., Tahara T. (2022). Scanning two-dimensional fluorescence lifetime correlation spectroscopy: Conformational dynamics of DNA Holliday junction from microsecond to subsecond. J. Phys. Chem. Lett..

[16] Zeug A., Woehler A., Ponimaskin E.G. (2012). Quantitative intensity-based fret approaches—a comparative snapshot. Biophys. J..

[17] Kuppa S., Deveryshetty J., Antony E. (2022). Rtt105 regulates rpa function by configurationally stapling the flexible domains. Nat. Commun..

[18] Kilic Z., Sgouralis I., Pressé S. (2021). Generalizing hmms to continuous time for fast kinetics: hidden markov jump processes. Biophys. J..

[19] Kapusta P., Wahl M., Enderlein J. (2007). Fluorescence lifetime correlation spectroscopy. J. Fluoresc..

[20] Ishii K., Tahara T. (2013). Two-dimensional fluorescence lifetime correlation spectroscopy. 1. principle. J. Phys. Chem. B.

[21] Otosu T., Ishii K., Tahara T. (2015). Microsecond protein dynamics observed at the single-molecule level. Nat. Commun..

[22] Yoo J., Kim J.-Y., Chung H.S. (2020). Fast three-color single-molecule FRET using statistical inference. Nat. Commun..

[23] Lerner E., Ingargiola A., Weiss S. (2018). Characterizing highly dynamic conformational states: The transcription bubble in RNAP-promoter open complex as an example. J. Chem. Phys..

[24] Saurabh A., Safar M., Pressé S. (2022). Single photon smFRET. I. theory and conceptual basis. bioRxiv.

[25] Sgouralis I., Pressé S. (2017). An introduction to infinite HMMs for single-molecule data analysis. Biophys. J..

[26] Sgouralis I., Madaan S., Pressé S. (2019). A Bayesian nonparametric approach to single molecule Förster resonance energy transfer. J. Phys. Chem. B.

[27] Fox E.B., Sudderth E.B., Willsky A.S. (2011). A sticky HDP-HMM with application to speaker diarization. Ann. Appl. Stat..

[28] Teh Y.W., Jordan M.I., Blei D.M. (2006). Hierarchical Dirichlet processes. J. Am. Stat. Assoc..

[29] Jayaram S. (1994). A constructive definition of Dirichlet priors. Stat. Sin..

[30] Pitman J. (2002). Poisson–Dirichlet and GEM invariant distributions for split-and-merge transformations of an interval partition. Combinator. Probab. Comput..

[31] Ferguson T.S. (1973). A Bayesian analysis of some nonparametric problems. Ann. Stat..

[32] Gershman S.J., Blei D.M. (2012). A tutorial on Bayesian nonparametric models. J. Math. Psychol..

[33] Sgouralis I., Whitmore M., Pressé S. (2018). Single molecule force spectroscopy at high data acquisition: A Bayesian nonparametric analysis. J. Chem. Phys..

[34] Tavakoli M., Taylor J.N., Pressé S. (2017). Single molecule data analysis: An introduction. Adv. Chem. Phys..

[35] Tavakoli M., Jazani S., Pressé S. (2020). Pitching single-focus confocal data analysis one photon at a time with Bayesian nonparametrics. Phys. Rev. X.

[36] Tavakoli M., Jazani S., Pressé S. (2020). Direct photon-by-photon analysis of time-resolved pulsed excitation data using Bayesian nonparametrics. Cell Rep. Phys. Sci..

[37] Bryan J.S., Sgouralis I., Pressé S. (2022). Diffraction-limited molecular cluster quantification with Bayesian nonparametrics. Nat. Comput. Sci..

[38] Fazel M., Jazani S., Pressé S. (2022). High resolution fluorescence lifetime maps from minimal photon counts. ACS Photonics.

[39] Okamoto K., Sako Y. (2016). State transition analysis of spontaneous branch migration of the Holliday junction by photon-based single-molecule fluorescence resonance energy transfer. Biophys. Chem..

[40] Hohng S., Joo C., Ha T. (2004). Single-molecule three-color FRET. Biophys. J..

[41] McKinney S.A., Déclais A.C., Ha T. (2003). Structural dynamics of individual Holliday junctions. Nat. Struct. Biol..

[42] McKinney S.A., Freeman A.D.J., Ha T. (2005). Observing spontaneous branch migration of Holliday junctions one step at a time. Proc. Natl. Acad. Sci. USA.

[43] Panyutin I.G., Biswas I., Hsieh P. (1995). A pivotal role for the structure of the Holliday junction in DNA branch migration. EMBO J..

[44] Metropolis N., Rosenbluth A.W., Teller E. (1953). Equation of state calculations by fast computing machines. J. Chem. Phys..

[45] Hastings W.K. (1970). Monte Carlo sampling methods using Markov chains and their applications. Biometrika.

[46] Fazel M., Wester M.J., Lidke K.A. (2019). Bayesian multiple emitter fitting using reversible jump Markov chain Monte Carlo. Sci. Rep..

[47] Jazani S., Sgouralis I., Pressé S. (2019). An alternative framework for fluorescence correlation spectroscopy. Nat. Commun..

[48] Rabiner L.R. (1989). A tutorial on hidden Markov models and selected applications in speech recognition. Proc. IEEE.

[49] Fazel M., Alexander V., Pressé S. (2022). Fluorescence lifetime: beating the irf and interpulse window. bioRxiv.

[50] Gillespie D.T. (1976). A general method for numerically simulating the stochastic time evolution of coupled chemical reactions. J. Comput. Phys..

[51] Kilic Z., Sgouralis I., Pressé S. (2021). Extraction of rapid kinetics from smFRET measurements using integrative detectors. Cell Rep. Phys. Sci..

[52] Karymov M., Daniel D., Lyubchenko Y.L. (2005). Holliday junction dynamics and branch migration: single-molecule analysis. Proc. Natl. Acad. Sci. USA.

[53] Ferapontova E.E., Mountford C.P., Mount A.R. (2008). Electrochemical control of a DNA Holliday Junction nanoswitch by Mg2+ ions. Biosens. Bioelectron..

[54] Pirchi M., Tsukanov R., Nir E. (2016). Photon-by-photon hidden Markov model analysis for microsecond single-molecule FRET kinetics. J. Phys. Chem. B.

[55] Kilic Z., Sgouralis I., Pressé S. (2021). Generalizing HMMs to continuous time for fast kinetics: Hidden Markov jump processes. Biophys. J..

[56] Sebolt-Leopold J.S., English J.M. (2006). Mechanisms of drug inhibition of signalling molecules. Nature.

[57] Jazani S., Sgouralis I., Pressé S. (2019). A method for single molecule tracking using a conventional single-focus confocal setup. J. Chem. Phys..

