# Supplementary material for: Single-photon smFRET. III. Application to pulsed illumination
Source: Biophys Rep (N Y). 2022 Nov 25;2(4):100088. doi: 10.1016/j.bpr.2022.100088 (PMC9747580; doi:10.1016/j.bpr.2022.100088)
Supplement: Document S2, Article plus supporting material [file mmc2.pdf]

Biophysical Reports

ARTICLE

Single-photon smFRET. III. Application to pulsed illumination

Matthew Safar,[1,2] Ayush Saurabh,[1,3] Bidyut Sarkar,[4,5] Mohamadreza Fazel,[1,3] Kunihiko Ishii,[4,5] Tahei Tahara,[4,5] Ioannis Sgouralis,[6] and Steve Pressé[1,3,7,*]

[1]Center for Biological Physics, Arizona State University, Tempe, Arizona; [2]Department of Mathematics and Statistical Science, Arizona State University, Tempe, Arizona; [3]Department of Physics, Arizona State University, Tempe, Arizona; [4]Molecular Spectroscopy Laboratory, RIKEN, 2-1 Hirosawa, Wako, Saitama, Japan; [5]Ultrafast Spectroscopy Research Team, RIKEN Center for Advanced Photonics (RAP), 2-1 Hirosawa, Wako, Saitama, Japan; [6]Department of Mathematics, University of Tennessee Knoxville, Knoxville, Tennessee; and [7]School of Molecular Sciences, Arizona State University, Phoenix, Arizona

ABSTRACT Förster resonance energy transfer (FRET) using pulsed illumination has been pivotal in leveraging lifetime information in FRET analysis. However, there remain major challenges in quantitative single-photon, single-molecule FRET (smFRET) data analysis under pulsed illumination including 1) simultaneously deducing kinetics and number of system states; 2) providing uncertainties over estimates, particularly uncertainty over the number of system states; and 3) taking into account detector noise sources such as cross talk and the instrument response function contributing to uncertainty; in addition to 4) other experimental noise sources such as background. Here, we implement the Bayesian nonparametric framework described in the first companion article that addresses all aforementioned issues in smFRET data analysis specialized for the case of pulsed illumination. Furthermore, we apply our method to both synthetic as well as experimental data acquired using Holliday junctions.

WHY IT MATTERS?

In the first companion article of this series, we developed new methods to analyze noisy single-molecule Förster resonance energy transfer data. These methods eliminate the requirement of a priori specifying the dimensionality of the physical model describing a molecular complex's kinetics. Here, we apply these methods to experimentally obtained datasets with samples illuminated by laser pulses at regular time intervals. In particular, we study conformational dynamics of Holliday junctions.

INTRODUCTION

Among the many fluorescence methods available (1–7), single-molecule Förster resonance energy transfer (smFRET) has been useful in probing interactions and conformational changes on nanometer scales (8–12). This is typically achieved by estimating FRET efficiencies (and system states) at all instants of an smFRET trace and subsequently estimating transition rates. Furthermore, among different FRET modalities, FRET efficiencies are most accurately determined under pulsed illumination (13–15), where the FRET dyes are illuminated by short laser bursts at known times.

Under this illumination procedure, photon arrival times are recorded with respect to the immediately preceding pulse, thereby facilitating an accurate estimation of fluorescence lifetimes as well as FRET rates. As such, in this article, we will focus on single-photon smFRET analysis under pulsed illumination.

Under pulsed illumination, information on kinetic parameters present in smFRET data is traditionally learned by binned photon methods, thereby eliminating lifetime information altogether (16–18); bulk correlative methods (19–21); and single-photon methods (14,22,23). However, these methods are parametric, i.e., require fixing the number of system states a priori, and necessarily only learn system kinetics even though information on the number of system states is encoded in the data.

In this article, we implement a general smFRET analysis framework that was presented in Sec. 2.5.1 of the first companion manuscript (24) for the case of pulsed illumination to learn full distributions. In other words, probability distributions over parameters take into account uncertainties from all existing sources such as

Submitted July 21, 2022, and accepted for publication November 21, 2022.

*Correspondence: spresse@asu.edu

Editor: Jorg Enderlein.

https://doi.org/10.1016/j.bpr.2022.100088

© 2022 The Authors.

This is an open access article under the CC BY license (http://creativecommons.org/licenses/by/4.0/).

cross talk and background. These parameters include the system transition probabilities and photophysical rates, that is, donor and acceptor relaxation and FRET rates, with special attention paid to uncertainty arising from sources such as inherent stochasticity in photon arrival times and detectors. As our main concern is deducing the number of system states using single-photon arrivals while incorporating detector effects, we leverage the formalism of infinite hidden Markov models (iHMMs) (25–30) within the Bayesian nonparametric (BNP) paradigm (25,26,31–38). The iHMM framework assumes an a priori infinite number of system states with associated transition probabilities, where the number of system states warranted by input data is enumerated by those states most visited over the course of the system state trajectory.

Next, to benchmark our BNP-FRET sampler, we analyzed synthetic and experimental smFRET data acquired using a single confocal microscope with pulsed illumination optimized to excite donor dyes.

In particular, we employ a broad range of experimental data acquired from Holliday junctions (HJs) with an array of different kinetic rates due to varying buffer concentration of $MgCl_2$ (39–42).

MATERIALS AND METHODS

Terminology convention

To be consistent throughout our three-part article, we precisely define some terms as follows:

1. A macromolecular complex under study is always referred to as a system.
2. The configurations through which a system transitions are termed system states, typically labeled using σ.
3. FRET dyes undergo quantum mechanical transitions between photophysical states, typically labeled using ψ.
4. A system-FRET combination is always referred to as a composite.
5. A composite undergoes transitions among its superstates, typically labeled using φ.
6. All transition rates are typically labeled using λ.
7. The symbol N is generally used to represent the total number of discretized time windows, typically labeled with n.
8. The symbol w_n is generally used to represent the observations in the n-th time window.

Forward model and inverse strategy

In this section, we first briefly illustrate the adaptation of the general formalism described in our first companion article (24) to the pulsed illumination case. Next, we present a specialized inference procedure. The details of the framework not provided herein can be found in the supporting material.

As before, we consider a molecular complex labeled with a donor-acceptor FRET pair. As the molecular complex transitions through its M_σ system states indexed by $\sigma_{1:M_\sigma}$, laser pulses (optimized to excite the donor) separated by time τ may excite either the donor or acceptor to drive transitions among the photophysical states, $\psi_{1:M_\psi}$, as defined in the first companion article (24). Such photophysical transitions lead to photon emissions that may be detected in either donor or acceptor channels. The set of N observations, e.g., photon arrival times, from N pulses are recorded as

$$\mathbf{w} = \{w_1, w_2, \ldots, w_N\}. \tag{1}$$

Here, each individual measurement is a pair $w_n = (\mu_n^d, \mu_n^a)$, where μ_n^d and μ_n^a are the recorded arrival times (also known as microtimes) after the n-th pulse in both donor and acceptor channels, respectively. In cases where there is no photon detection, we denote the absent microtimes with $\mu_n^d = \varnothing$ and $\mu_n^a = \varnothing$ for donor and acceptor channels, respectively.

As is clear from Fig. 1, smFRET traces are inherently stochastic due to the nature of photon excitation, emission, and noise introduced by detector electronics. To analyze such stochastic systems, we begin with the most generic likelihood derived in Eq. 51 of the first companion article (24),

$$L \propto \boldsymbol{\rho}_{start} \mathbf{Q}_1 \ldots \mathbf{Q}_n \ldots \mathbf{Q}_N \boldsymbol{\rho}_{norm}^T, \tag{2}$$

where $\boldsymbol{\rho}_{start}$ is the initial probability vector for the system-FRET composite to be in one of $M_\phi = (M_\sigma \times M_\psi)$ superstates, and $\boldsymbol{\rho}_{norm}$ is a vector that sums the elements of the propagated probability vector. Here, we recall that $\mathbf{Q}_n$ is the transition probability matrix between pulses at t_n and t_{n+1}, characterizing system-FRET composite transitions among superstates.

The propagators $\mathbf{Q}_n$ above adopt different forms depending on whether a photon is detected or not during the associated period. Their most general forms are derived in the section on illumination features in the first companion article (24). However, these propagators involve computationally expensive integrals, and thus we make a few approximations here as follows: 1) we assume that the system state remains the same over an interpulse period since typical system kinetic timescales (typically 1 ms or more) are much longer than interpulse periods (≈ 100 ns) (41,43), and 2) the interpulse period (≈ 100 ns) is longer than the donor and acceptor lifetimes ($\approx$ a few ns) (41,43) such that they relax to the ground state before the

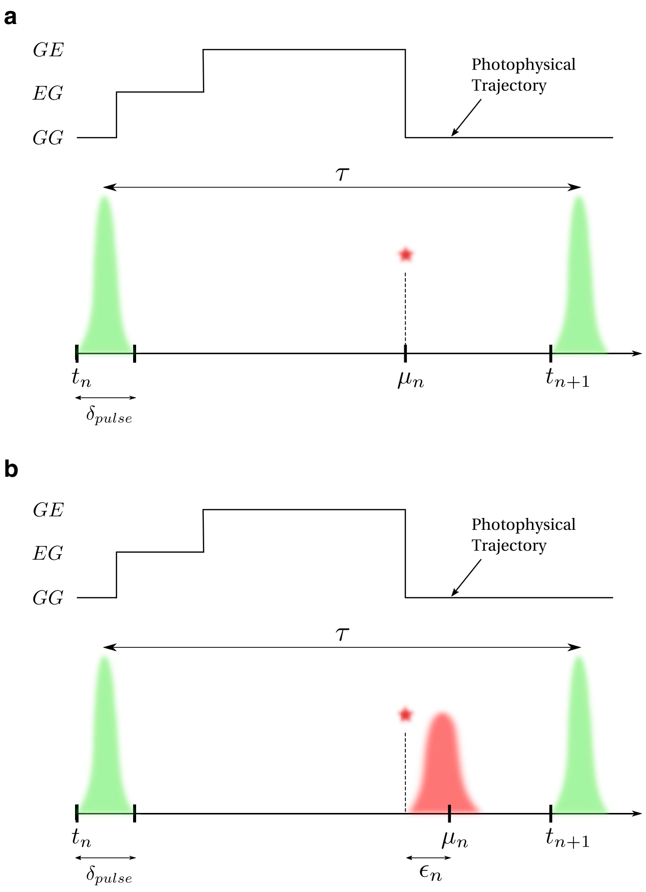

FIGURE 1 Events over a pulsed illumination experiment pulse window. Here, the beginning of the n-th interpulse window of size τ is marked by time t_n. The FRET labels originally in state GG (donor and acceptor, respectively, in ground states) are excited by a high intensity burst (shown in *green*) to the state EG (only donor excited) for a very short time, δ_{pulse}. If FRET occurs, the donor transfers its energy to the acceptor and resides in the ground state, leaving the FRET labels in the GE state (only acceptor excited). The acceptor then emits a photon to be registered by the detector at microtime μ_n. When using ideal detectors, the microtime is the same as the photon emission time as shown in (*a*). However, when the timing hardware has jitter (shown in *red*), a small delay ε_n is added to the microtime as shown in (*b*). For convenience, we have reproduced this figure from our first companion article (24).

next pulse. Furthermore, we will demonstrate a specialized sampling scheme under these physically motivated approximations.

The immediate implications of the first assumption are that the system transitions may now, to a good approximation, only occur at the beginning of each pulse. Consequently, the evolution of the FRET pair between two consecutive pulses is now exclusively photophysical, as the system state remains the same during interpulse times. As such, the system now evolves in equally spaced discrete time steps of size τ, where the system state trajectory can be written as

$$s_{1:N} = \{s_1, s_2, \ldots, s_n, \ldots, s_{N-1}, s_N\},$$

where s_n is the system state between pulses n and $n+1$. The stochastic evolution of the system states in such discrete steps is then determined by the transition probability matrix designated by $\mathbf{\Pi}_\sigma$. For example, in the simplest case of a molecular complex with two system states $\sigma_{1:2}$, this matrix is computed as follows:

$$\begin{aligned} \mathbf{\Pi}_\sigma &= \mathbf{exp}\left(\tau\begin{bmatrix} * & \lambda_{\sigma_1\to\sigma_2} \\ \lambda_{\sigma_2\to\sigma_2} & * \end{bmatrix}\right) \\ &= \begin{bmatrix} \pi_{\sigma_1\to\sigma_1} & \pi_{\sigma_1\to\sigma_2} \\ \pi_{\sigma_2\to\sigma_1} & \pi_{\sigma_2\to\sigma_2} \end{bmatrix}, \end{aligned} \tag{3}$$

where the matrix in the exponential contains transition rates among the system states and the $*$ represents the negative row sum.

Next, by assumption two, we can further suppose that the fluorophores always start in the ground state at the beginning of every pulse. As a result, we treat pulses independently and write the probability of observation w_n as

$$p(w_n|s_n, \mathbf{G}_\psi) = \boldsymbol{\rho}_{ground}\mathbf{Q}_n^\psi(s_n)\boldsymbol{\rho}_{norm}^T, \tag{4}$$

where $\boldsymbol{\rho}_{ground}$ denotes the probability vector when the FRET pair is in the ground state at the beginning of each pulse, $\mathbf{G}_\psi$ is the generator matrix with only photophysical transition rates, and $\mathbf{Q}_n^\psi(s_n)$ is the photophysical propagator for the n-th interpulse period.

We further organize the observation probabilities of Eq. 4 into a newly defined detection matrix $\mathbf{D}_n^\sigma$ with its elements given by $(\mathbf{D}_n^\sigma)_{s_n\to\sigma_j} = p(w_n|s_n, \mathbf{G}_\psi)$. Here, we note that the index j does not appear on the right-hand side because the system state does not change during an interpulse window, resulting in the independence of observation probability from the next system state, s_{n+1}. The explicit formulas for the observation probabilities are provided in the supporting material.

Now, using the matrix $\mathbf{D}_n^\sigma$, we define the reduced propagators for each interpulse period as

$$\mathbf{\Pi}_n^\sigma = \mathbf{\Pi}_\sigma \odot \mathbf{D}_n^\sigma, \tag{5}$$

where $\odot$ denotes the element-by-element product.

Finally, using these simplified propagators, we can write the likelihood for an smFRET trace under pulsed illumination as

$$L = p(\mathbf{w}|\boldsymbol{\rho}_{start}, \mathbf{\Pi}_\sigma, \mathbf{G}_\psi) \propto \boldsymbol{\rho}_{start}\mathbf{\Pi}_1^\sigma\mathbf{\Pi}_2^\sigma\ldots\mathbf{\Pi}_N^\sigma\boldsymbol{\rho}_{norm}^T, \tag{6}$$

as also introduced in the section on illumination features in the first companion article (24). This form of the likelihood is advantageous in that it allows empty pulses to be computed as a simple product, greatly reducing computational cost.

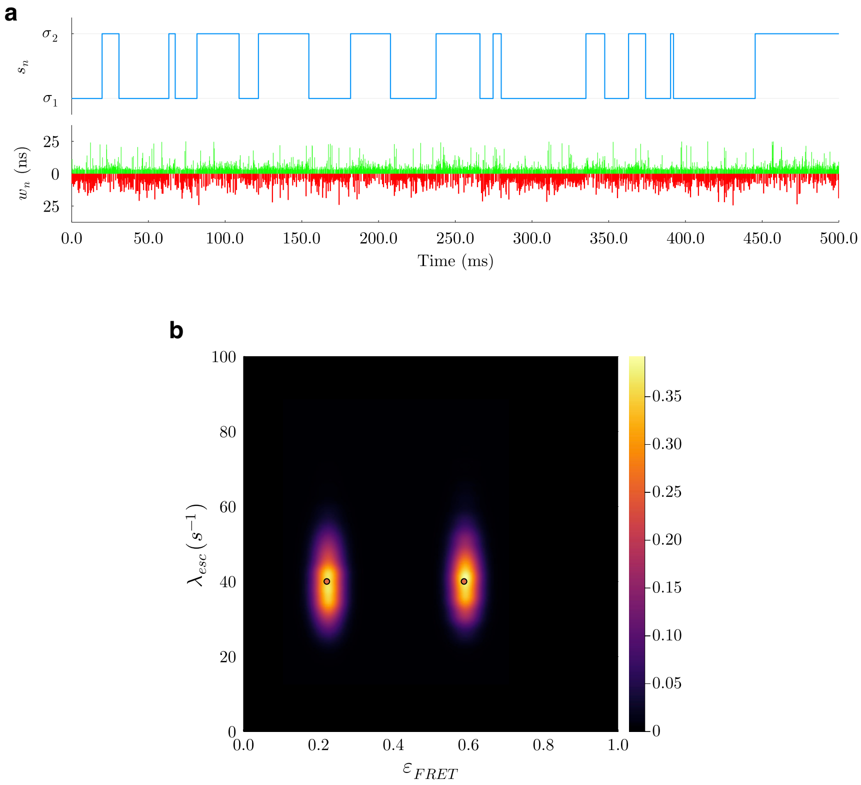

FIGURE 2 Analysis on synthetic data for a system with two system states. In (*a*), we show a section of synthetic data produced with the values in Table S2. Furthermore, the system state trajectory is shown in blue. Below this, the arrival times of donor and acceptor photons μ_n^d and μ_n^a are shown in green and red, respectively. In (*b*), we plot the bivariate distribution over escape rates and FRET efficiencies. The ground truth is shown with red dots corresponding to an escape rate of 40 s^{-1} and FRET efficiencies of 0.22 and 0.59. λ_{esc} ε_{FRET}. As seen, the BNP-FRET sampler clearly distinguishes two system states with maximum a posteriori (MAP) estimates for the associated escape rates of $\approx 38^{+7}_{-7}$ and $\approx 40^{+7}_{-7}$ s^{-1} and for FRET efficiencies of $\approx 0.21^{+0.03}_{-0.03}$ and $\approx 0.59^{+0.03}_{-0.03}$. We have smoothed the distributions using kernel density estimation for illustration purposes only.

In the following, we first illustrate a parametric inference procedure assuming a given number of system states. We next generalize the procedure developed to the nonparametric case to deduce the number of system states along with the rest of parameters.

Inference procedure: Parametric sampler

With the likelihood at hand, we construct the posterior as follows

$$p(\boldsymbol{\rho}_{start}, \boldsymbol{\Pi}_\sigma, \mathbf{G}_\psi | \mathbf{w}) \propto p(\mathbf{w} | \boldsymbol{\rho}_{start}, \boldsymbol{\Pi}_\sigma, \mathbf{G}_\psi) p(\boldsymbol{\rho}_{start}) p(\mathbf{G}_\psi) p(\boldsymbol{\Pi}_\sigma), \tag{7}$$

where we assume that the unknown parameters, including the initial probability vector, $\boldsymbol{\rho}_{start}$, the photophysical transition rates in the generator matrix $\mathbf{G}_\psi$, and the transition probabilities among system states in propagator $\boldsymbol{\Pi}_\sigma$ are independent, allowing us to conveniently write the prior on these parameters as a product (the last three terms on right hand side). Here, we can sample the set of unknowns using the above posterior with the Gibbs sampling procedure described in the first companion article (see the section describing inverse strategy in (24)). However, a computationally more convenient inference procedure that allows direct sampling is accomplished by writing the posterior of Eq. 7 as a marginalization (sum) over state trajectories as follows

$$\begin{aligned} p(\boldsymbol{\rho}_{start}, \boldsymbol{\Pi}_\sigma, \mathbf{G}_\psi | \mathbf{w}) &= \sum_{s_{1:N}} p(\boldsymbol{\rho}_{start}, \boldsymbol{\Pi}_\sigma, \mathbf{G}_\psi, s_{1:N} | \mathbf{w}) \\ &\propto \sum_{s_{1:N}} p(\mathbf{w} | \boldsymbol{\Pi}_\sigma, \mathbf{G}_\psi, s_{1:N}) p(\boldsymbol{\rho}_{start}) p(\mathbf{G}_\psi) p(\boldsymbol{\Pi}_\sigma) p(s_{1:N} | \boldsymbol{\rho}_{start}, \boldsymbol{\Pi}_\sigma), \end{aligned} \tag{8}$$

where $s_{1:N} = \{s_1, s_2 \ldots, s_N\}$ denotes a system state trajectory. Now, we can use the nonmarginal posterior

$$\begin{aligned} p(\boldsymbol{\rho}_{start}, \boldsymbol{\Pi}_\sigma, \mathbf{G}_\psi, s_{1:N} | \mathbf{w}) \propto\; & p(\mathbf{w} | \boldsymbol{\Pi}_\sigma, \mathbf{G}_\psi, s_{1:N}) p(\boldsymbol{\rho}_{start}) \\ & p(\mathbf{G}_\psi) p(\boldsymbol{\Pi}_\sigma) p(s_{1:N} | \boldsymbol{\rho}_{start}, \boldsymbol{\Pi}_\sigma) \end{aligned} \tag{9}$$

to sample the trajectory $s_{1:N}$, which, in turn, allows direct sampling of the elements of propagator $\mathbf{\Pi}_\sigma$ described shortly. For priors on $\boldsymbol{\rho}_{start}$ and rates in $\mathbf{G}_\psi$, we, respectively, use Dirichlet and Gamma distributions similar to Eqs. 65 and 66 of the first companion article (24). We sample the system state trajectory $s_{1:N}$ by recursively sampling the states using a forward filtering backward sampling algorithm described in section S4.3.

Finally, for each row in the propagator $\mathbf{\Pi}_\sigma$, we use a Dirichlet prior

$$\pi_m \sim \textbf{Dirichlet}(\alpha\boldsymbol{\beta}), m = 1, 2, \ldots, M_\sigma, \qquad (10)$$

where M_σ is the number of system states and $\boldsymbol{\pi}_m$ denotes the m-th row of the propagator. Here, the hyperparameters α and $\boldsymbol{\beta}$ are, respectively, the concentration parameter and a vector of length M_σ described in the first companion article (see Section 3.2.2 of (24)). We can now directly generate samples for the transition probability vectors $\boldsymbol{\pi}_m$ of length M_σ via prior-likelihood conjugacy as (see section S4.3)

$$\pi_m \sim \textbf{Dirichlet}(\mathbf{n}_m + \alpha\boldsymbol{\beta}), m = 1, 2, \ldots, M_\sigma^{\max},$$

where the vector $\mathbf{n}_m$ collects the number of times each transition out of system state σ_m occurs obtained using the system state trajectory.

After constructing the posterior, we can make inferences on the parameters by drawing samples from the posterior. However, as the resulting posterior has a nonanalytical form, it cannot be directly sampled. Therefore, we develop a Markov chain Monte Carlo sampling procedure (37,38,44–47) to draw samples from the posterior.

Our Markov chain Monte Carlo sampling scheme follows a Gibbs sampling technique, sweeping through updates of the set of parameters in the following order: 1) photophysical transition rates including donor relaxation rates λ_d (inverse of donor lifetime), acceptor relaxation rate λ_a (inverse of acceptor lifetime), FRET rates $\lambda^{FRET}_{\sigma_{1:M_\sigma}}$ for each system state, and excitation rate (inverse of excitation probability π_{ex}) using the Metropolis-Hastings(MH) step; 2) transition probabilities between system states, $\pi_{1:M_\sigma}$, by directly drawing samples from the posterior; 3) the system states trajectory, $\mathscr{S}$, using a forward-backward sampling procedure (48); and 4) the initial probabilities, $\boldsymbol{\rho}_{start}$, by taking direct samples. In the end, the chains of samples drawn can be used for subsequent numerical analysis.

Inference procedure: Nonparametrics sampler

The smFRET data analysis method illustrated above assumes a given number of system states, M_σ. However, in many applications, the number of system states is not specified a priori. Here, we describe a generalization of our parametric method to address this shortcoming and estimate the number of system states simultaneously along with other unknown parameters.

We accomplish this by modifying our previously introduced parametric posterior as follows. First, we suppose an infinite number of system states $(M_\sigma \to \infty)$ for the likelihood introduced previously and learn the transition matrix $\mathbf{\Pi}_\sigma$. The number of system states can then be interpreted as those appreciably visited over the course of the trajectory.

To incorporate this infinite system state space into our inference strategy, we leverage the iHMMs (25,26,28–30) from the BNP repertoire, placing a hierarchical Dirichlet process prior over the infinite set of system states as described in the first companion article (the inverse strategy section in (24)). However, as detailed in the first companion manuscript (the inverse strategy section in (24)), dealing with an infinite number of random variables, though feasible, is not computationally efficient, and we approximate this infinite value with a large number, $M_\sigma^{\max}$, reducing our hierarchical Dirichlet process prior to

$$\boldsymbol{\beta} \sim \textbf{Dirichlet}\left(\frac{\gamma}{M_\sigma^{\max}}, \ldots, \frac{\gamma}{M_\sigma^{\max}}\right),$$

$$\boldsymbol{\pi}_m \sim \textbf{Dirichlet}(\alpha\boldsymbol{\beta}), m = 1, \ldots, M_\sigma^{\max}.$$

Here, $\boldsymbol{\beta}$ denotes the base probability vector of length $M_\sigma^{\max}$ serving itself as a prior on the probability transition matrix $\mathbf{\Pi}_\sigma$, and $\boldsymbol{\pi}_m$ is the m-th row of $\mathbf{\Pi}_\sigma$. Moreover, γ is a positive scalar hyperparameter of the Dirichlet process prior often chosen to be one. As such, we ascribe identical weights across the state space a priori for computational convenience (28,29,49).

Now, equipped with the nonparametric posterior, we proceed to simultaneously make inferences on transition probabilities, excited-state escape rates, and the remaining parameters. To do so, we employ the Gibbs sampling scheme detailed in the inverse strategy section in the first companion article (24), except that we must now also sample the system state trajectory $s_{1:N}$. More details on the overall sampling scheme are found in section S4 of the supporting material.

RESULTS

The main objective of our method is to learn full distributions over 1) transition probabilities among $M_\sigma^{\max}$ system states determining, in turn, the corresponding system transition rates and the effective number of system states, and 2) photophysical transition rates,

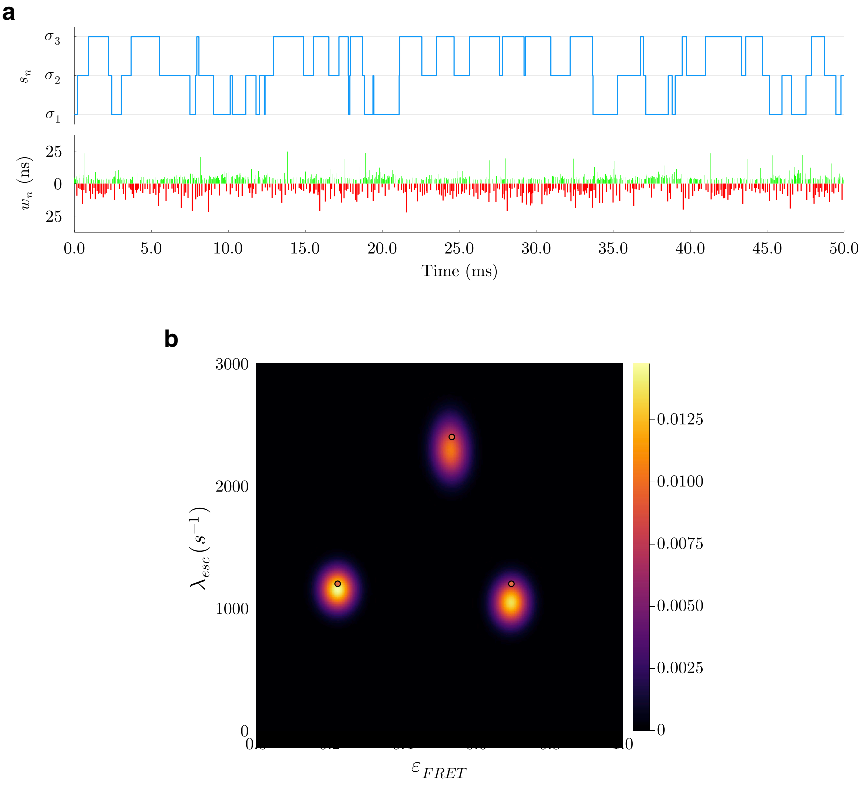

FIGURE 3 Analysis on synthetic data for three system states. In (*a*), we have a section of synthetic data produced with the values from Table S3. The system state trajectory is seen in blue. Below this, the arrival times of donor and acceptor photons μ_n^d and μ_n^a are shown in green and red, respectively. In (*b*), we plot the distribution over escape rates and FRET efficiencies ε_{FRET}. The red dots show ground truths corresponding to escape rates of 1,200, 2,400, and 1,200 s^{-1} and FRET efficiencies of 0.22, 0.53, and 0.7. From our maximum a posteriori (MAP) estimate, λ_{esc} ε_{FRET} we clearly see three system states with escape rates of $1,100^{+60}_{-60}$, $2,300^{+131}_{-128}$, and $1,050^{+80}_{-80}$ s^{-1}.

including FRET rates $\lambda_{1:M}^{FRET}$, and fluorophores' relaxation rates (inverse of lifetimes) λ_a and λ_d.

To sample from distributions over these parameters, the BNP-FRET sampler requires input data comprised of photon arrival time traces from both donor and acceptor channels as well as a set of precalibrated input parameters including camera effects such as cross talk matrix and detection efficiency (see Sec. 2.4 and example V of the first companion article (24)); background emission (see the section on background in the first companion article and Section S2.4); and the instrument response function (IRF) (see illumination features sectoin in the first companion article (24) and Section S2.3).

Here, we first show that our method samples posteriors over a set of parameters employing realistic synthetic data generated using the Gillespie algorithm (50) to simulate system and photophysical transitions while incorporating detector artefacts such as crosstalk (see the synthetic data generation section in the first companion article (24)). The list of parameters used in data generation for all figures is provided in Section S6. Furthermore, prior hyperparameters used in the analysis of synthetic and experimental data are listed in Section S3.

We first show that our method works for the simplest case of slow transitions compared with the interpulse period (25 ns) with two system states using synthetic data (see Fig. 2). Next, we proceed to tackle more challenging synthetic data with three system states and higher transition rates (Fig. 3). We show that our nonparametric algorithm correctly infers system transition probabilities and thus the number of system states (see Fig. 3).

After demonstrating the performance of our method using synthetic data, we use experimental data to investigate the kinetics of HJs under different $MgCl_2$ concentrations in buffer (see Fig. 4).

Simulated data analysis

To help validate BNPs on smFRET single-photon data, we start with a simple case of a two-state system and select kinetics similar to those of the experimental data sets, c.f., the HJ in 10 mM $MgCl_2$, with escape rates of 40 s^{-1} for both system states (51). The generated system state trajectory and photon traces over a period of 500 ms from both channels are shown in Fig. 2 *a*.

Fig. 2 *b* shows the bivariate posterior distribution over FRET efficiencies, ε_{FRET}, defined as $\varepsilon_{FRET} = \lambda_{FRET}/(\lambda_{FRET} + \lambda_d)$, and system escape rates, i.e., obtained by computing the logarithm of the propagator matrix, with two peaks corresponding to the two system states most visited by the sampler. Furthermore, the ground truths, designated by red dots, fall within the posterior with a relative error of less than

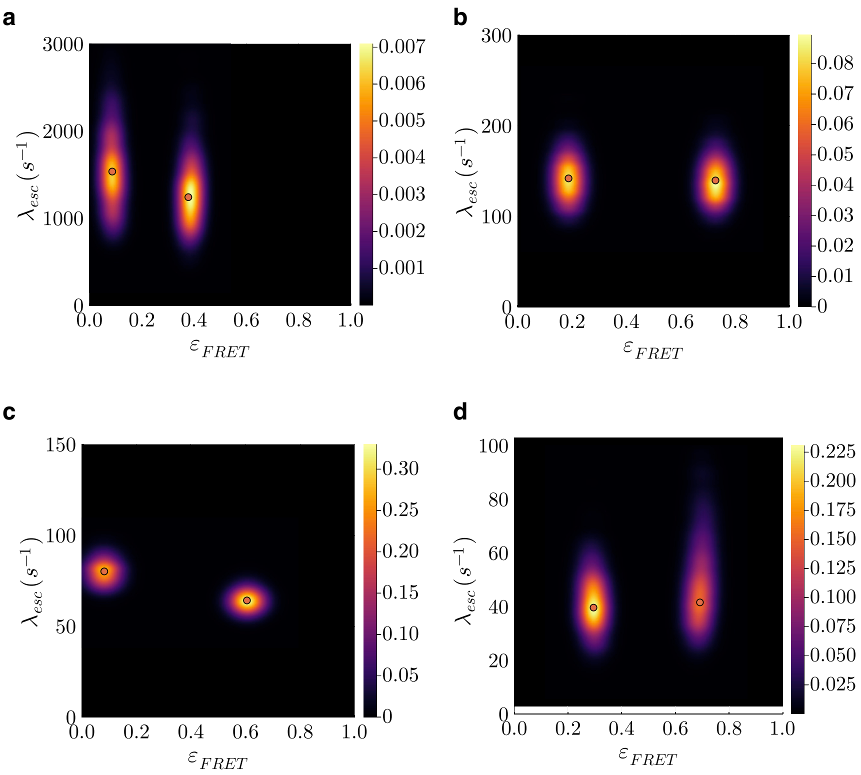

FIGURE 4 The bivariate posterior for the conformational transition rates λ_{esc} and FRET efficiencies ε_{FRET} for experimental data acquired in the presence of different HJ concentrations. Here, we show our bivariate posteriors where red dots show MAP estimates. In (*a*), we show the posterior for a sample with 1 mM $MgCl_2$. We report escape rates of $1,530^{+500}_{-550}$ and $1,240^{+420}_{-420}$ s^{-1} in this case. The posterior for a sample with 3 mM $MgCl_2$ is shown in (*b*). We report escape rates of 140^{+38}_{-38} and 142^{+32}_{-32} s^{-1} for this case. In (*c*), we show our posterior for a sample with 5 mM $MgCl_2$. Here, we report escape rates of 64^{+9}_{-9} and 80^{+10}_{-10} s^{-1}. The posterior in (*d*) is for a sample with 10 mM $MgCl_2$. We report escape rates of 39^{+17}_{-12} and 41^{+23}_{-12} s^{-1}.

3% from the posterior modes. The results for the remaining parameters, including donor and acceptor transition rates, FRET transition rates, and system transition probabilities, are presented in Section S7.

To showcase the critical role played by BNPs, we also consider the more difficult case of a sample with three system states and faster system state kinetics ranging over 1,200–2,500 s^{-1}. We do so by simulating photon traces in both donor and acceptor channels over a period of $\sim$150 ms. A 50 ms section of the synthetic photon trace is shown in Fig 3 *a*.

Using direct photon arrivals from the generated photon trace, we find that the most probable system state trajectories sampled by BNP-FRET visit the correct number of system states, as shown in Fig 3 *b*, while inferring all other parameters. Furthermore, the BNP-FRET sampler estimates the system transition rates and thus the escape rates (i.e., sum of transition rates out of a given state) where the ground-truth escape rates differ from the posterior peaks by a relative average error of less than 8%. The results for the remaining parameters are provided in Section S7.

Experimental data analysis: HJ

In this section, we benchmark our method over a wide range of kinetic rates employing experimental data acquired using HJ under varying buffer $MgCl_2$ concentrations (15,51).

HJs are four-way double-helical DNA junctions existing in various structural configurations (41,52,53). When not interacting with multivalent metal ions, electrostatic repulsion between negatively charged phosphate groups of the four helical arms forces HJs to assume a wide configuration where the arms lie along the two diagonals of a square. However, in the presence of ions, such as Mg^{2+}, interaction with the phosphate groups results in electrostatic screening. This reduced repulsion induces transitions to what is believed to be primarily two compact stacked configurations/conformations. The transitions between both conformations necessitates passing through the intermediate open configuration. Since, at high ion concentrations, displacing ions away from the phosphate group becomes increasingly difficult, in this scenario, we anticipate smaller transition rates between both conformations.

The HJ kinetic rates have been studied using both fluorescence lifetime correlation spectroscopy (15) and HMM analysis (54) on diffusing HJs assuming a priori a pair of high and low FRET system states. As expected, these previous studies show kinetic rates decreasing with increasing $MgCl_2$ concentrations (41,43) and correspondingly longer dwells.

Here, our method, free from averaging and binning that are otherwise common in HMM analysis, is particularly well suited to learn the rapid kinetics at low Mg^{2+} concentrations. We apply our BNP-FRET to data

acquired from HJs at 1, 3, 5, and 10 mM $MgCl_2$ concentrations and sample the photophysical transition rates and the system transition probabilities.

The acquired bivariate posterior distributions over the FRET efficiencies and escape rates (computed via the logarithm of the system transition probability matrix $\mathbf{\Pi}_\sigma$) are presented in Fig. 4. Moreover, estimates for the other parameters can be found in Section S7. We note that our results are obtained on a single-molecule basis with a photon budget of 10^4–10^5 photons.

For all four concentrations (see Fig. 4), our BNP-FRET sampler most frequently visited only two system states, while this was given as an input to the other analysis methods (15,54). Moreover, both escape rates are found to have similar values with an average of approximately 1,400s^{-1} (1 mM $MgCl_2$), 140s^{-1} (3 mM $MgCl_2$), 72s^{-1} (5 mM $MgCl_2$), and 41 s^{-1} (10 mM $MgCl_2$). These escape rates are in close agreement with values reported by fluorescence lifetime correlation spectroscopy and H2MM methods (15,54) of $\approx 1,300$ s^{-1} (1 mM $MgCl_2$), ≈ 170 (3 mM $MgCl_2$), ≈ 100 (5 mM $MgCl_2$), and ≈ 60 s^{-1} (10 mM $MgCl_2$), which lie well within the bounds of our posteriors shown in Fig. 4 while simultaneously, and self-consistently, learning a number of system states.

Experimental data acquisition

In this section, we describe the protocol for preparing the surface-immobilized HJ sample labeled with a FRET pair and the procedure for recording smFRET traces from individual immobilized molecules. The sample preparation and recording of data follow previous work (55).

Sample preparation

The HJ used in this work consists of four DNA strands whose sequences are as follows:

R-strand: 5′-CGA TGA GCA CCG CTC GGC TCA ACT GGC AGT CG-3′

H-strand: 5′-CAT CTT AGT AGC AGC GCG AGC GGT GCT CAT CG-3′

X-strand: 5′-biotin-TCTTT CGA CTG CCA GTT GAG CGC TTG CTA GGA GGA GC-3′

B-strand: 5′-GCT CCT CCT AGC AAG CCG CTG CTA CTA AGA TG-3'

For surface immobilization, the X-strand was labeled with biotin at the 5′ end. For FRET measurements, the donor (ATTO-532) and acceptor (ATTO-647N) dyes were introduced into the H- and B-strands, respectively. In both cases, the dyes were labeled to thymine nucleotide at the 6th position from the 5′ ends of the respective strands (shown as T). All DNA samples (labeled or unlabeled) were purchased from JBioS (Shinjuku-ku, Japan) in the high-performance liquid chromatography purified form and were used without any further purification.

The HJ complex was prepared by mixing 1 mM solutions of R-, H-, B-, and X-strands in TN buffer (10 mM Tris-HCl with 50 mM NaCl, pH 8) at a 3:2:3:3 molar ratio, annealing the mixture at 94°C for 4 minutes, and gradually cooling it down (2°C–3°C min^{-1}) to room temperature (25°C). For smFRET measurements, we used a sample chamber (SecureSeal, GBL621502, Grace Bio-Labs, Bend, OR, USA) with a biotin-PEG-SVA (biotin-poly(ethylene glycol)-succinimidyl valerate)-coated coverslip. The chamber was first incubated with streptavidin (0.1 mg mL^{-1} in TN buffer) for 20 min. This was followed by washing the chamber with TN buffer (3 times) and injection of 1 nM HJ solution (with respect to its H-strand) for 3–10 s. After this incubation period, the chamber was rinsed with TN buffer (3 times) to remove unbound DNA, and it was filled with TN buffer containing 1 mM (or 5 mM) $MgCl_2$ and 2 mM Trolox for smFRET measurements.

smFRET measurements

The smFRET traces from individual HJs were recorded using a custom-built confocal microscope (Eclipse Ti, Nikon, Tokyo, Japan) equipped with the Perfect Focus System, a sample scanning piezo stage (Nano control B16-055), and a time-correlated single-photon counting module (SPC-130EM, Berlin, Germany).

The broadband light generated by a supercontinuum laser operating at 40 MHz (SC-400-4, Fianium, Southampton, UK) was filtered with a band-pass filter (FF01-525/30, Semrock, West Henrietta, NJ, USA) for exciting the donor dye, ATTO-532. This excitation light was introduced to the microscope using a single-mode optical fiber (P5-460B-PCAPC-1, Thorlabs, Newton, NJ, USA) and directed onto the sample using a dichroic mirror (ZT532/640rpc, Chroma, Cambridge, MA, USA) and a water immersion objective lens (Nikon Plan Apo IR 60×, numerical aperture: 1.27).

The excitation light was focused onto the top surface of the coverslip, and, during measurements, the focusing condition was maintained using the Perfect Focus System. The fluorescence signals were collected by the same objective, passed through the dichroic mirror, and guided to the detection assembly (Thorlabs DFM1/M) using a multimode fiber (Thorlabs M50L02S-A). Note that this multimode fiber (core diameter: 50 μm) also acts as the confocal pinhole. In the detection assembly, the fluorescence signals from the donor and acceptor dyes were separated using a dichroic mirror (ZT633rdc, Chroma Technology, Bellows Falls, VT, USA), filtered using band-pass filters (Chroma ET585/65m for donor and Semrock FF02-685/40 for

acceptor), and detected using separate hybrid detectors (Becker and Hickl HPM-100-40-C).

For each detected photon, its macrotime (absolute arrival time from the start of the measurement) was recorded with 25.2 ns resolution and its microtime (relative delay from the excitation pulse) was recorded with 6.1 ps resolution using the time-correlated single-photon counting module operating in time-tagging mode. A router (Becker and Hickl HRT-41) was used to process the signals from the donor and acceptor detectors.

For recording smFRET traces from individual HJs, we first imaged a 10 $\times$ 3 μm area of the sample using the piezo stage by scanning it linearly at a speed of 1 μm s^{-1} in the x direction and with an increment of 0.1 μm in the y direction. Individual HJs appeared as isolated bright spots in the image.

Next, we fitted the obtained donor and acceptor intensity images with multiple 2D Gaussian functions to determine the precise locations of individual HJs. Note that, during this image acquisition, the laser excitation power was kept to a minimum ($\sim$1 μW at the back aperture of the objective lens) to avoid photobleaching the dyes. In addition, we also employed an electronic shutter (Suruga Seiki, Shizuoka, Japan) in the laser excitation path to control the sample excitation as required.

Using the precise locations of individual HJs obtained, we recorded 30 s-long smFRET traces for each molecule by moving them to the center of the excitation beam using the piezo stage. For each trace, the laser excitation was blocked (using the shutter) for the first 5 s and was allowed to excite the sample for the remaining 25 s. Note that the smFRET traces were recorded using 40 μW laser excitation (at the back aperture of the objective lens) to maximize the fluorescence photons emitted from the dyes. We automated the process of acquiring smFRET traces from different molecules sequentially and executed it using a program written in house on Igor Pro (Wavemetrics, Portland, OR, USA).

DISCUSSION

The sensitivity of smFRET under pulsed illumination has been exploited to investigate many different molecular interactions and geometries (8–11,56). However, quantitative interpretation of smFRET data faces serious challenges including an unknown number of system states and robust propagation of uncertainty from noise sources such as detectors and background. These challenges ultimately mitigate our ability to determine full distributions over all relevant unknowns and, traditionally, have resulted in data pre- or postprocessing compromising the information otherwise encoded in the rawest form of data: single-photon arrivals.

Here, we provide a general BNP framework for smFRET data analysis starting from single-photon arrivals under a pulsed illumination setting. We simultaneously learn transition probabilities among system states as well as determine photophysical rates by incorporating existing sources of uncertainty such as background and cross talk.

We benchmark our method using both experimental and simulated data. That is, we first show that our method correctly learns parameters for the simplest case with two system states and slow system transition rates. Moreover, we test our method on more challenging cases with more than two states using synthetic data and obtain correct estimations for the system state transition probabilities and thus the number of system states along with the remaining parameters of interest. To further assess our method's performance, we analyzed experimental data from HJs suspended in solutions with a range of $\mathrm{MgCl_2}$ concentrations. These data were previously processed using other techniques assuming a fixed number of system states by binning photon arrival times (15).

Despite multiple advantages mentioned above for BNP-FRET, BNPs always come with an added computational cost as they take full advantage of information from single-photon arrival times and all existing sources of uncertainty. For this version of our general BNP method simplified for pulsed illumination, we further reduced the computational complexity by grouping empty pulses together. Therefore, the computational complexity increased only linearly with the number of input photons as the photons are treated independently.

The method described in this paper assumes a Gaussian IRF. However, the developed framework is not limited to a specific form for the IRF and can be used for data collected using any type of IRF by modifying Eq. 4. Furthermore, the framework is flexible in accommodating different illumination techniques such as alternating color pulses, which are typically used to directly excite the acceptor fluorophores. This can be achieved by simple modification of the propagator $\mathbf{Q}_n^{\psi}$ in Eq. 4. A future extension of this method could relax the assumption of a static sample by adding spatial dependence to the excitation rate as we explored in previous works (35,47,57). This would allow our method to learn the dynamics of diffusing molecules, as well as their photophysical and system state transition rates.

Code availability

The BNP-FRET software package is available on Github at https://github.com/LabPresse/BNP-FRET.

SUPPORTING MATERIAL

Supporting material can be found online at https://doi.org/10.1016/j.bpr.2022.100088.

ACKNOWLEDGMENTS

We thank Dr. Zeliha Kilic, Weiqing Xu, and J. Shephard Bryan IV for their contributions and insight into the project. We also thank Dr. Douglas Shepherd for providing insight into the workings of detectors and other experimental equipment and Irina Gopich for discussions on FRET likelihoods. T.T. acknowledges support from the JSPS KAKENHI (grant no. 18H05265). S.P. acknowledges support from the NIH NIGMS (R01GM130745) for supporting early efforts in nonparametrics and NIH NIGMS (R01GM134426) for supporting single-photon efforts.

DECLARATION OF INTERESTS

The authors declare no competing interests.

REFERENCES

1. Weiss, S. 1999. Fluorescence spectroscopy of single biomolecules. *Science.* 283:1676.
2. Lippincott-Schwartz, J., E. Snapp, and A. Kenworthy. 2001. Studying protein dynamics in living cells. *Nat. Rev. Mol. Cell Biol.* 2:444.
3. Huang, B., M. Bates, and X. Zhuang. 2009. Super-resolution fluorescence microscopy. *Annu. Rev. Biochem.* 78:993–1016.
4. Lelek, M., M. T. Gyparaki, …, C. Zimmer. 2021. Single-molecule localization microscopy. *Nat. Rev. Methods Primers.* 1:39.
5. Fazel, M., and M. J. Wester. 2022. Analysis of super-resolution single molecule localization microscopy data: A tutorial. *AIP Adv.* 12:010701.
6. Datta, R., T. M. Heaster, …, M. C. Skala. 2020. Fluorescence lifetime imaging microscopy: fundamentals and advances in instrumentation, analysis, and applications. *J. Biomed. Opt.* 25:1–43.
7. Garini, Y., I. T. Young, and G. McNamara. 2006. Spectral imaging: principles and applications. *Cytometry A.* 69:735–747.
8. Roy, R., S. Hohng, and T. Ha. 2008. A practical guide to single-molecule FRET. *Nat. Methods.* 5:507–516.
9. Mazal, H., and G. Haran. 2019. Single-molecule FRET methods to study the dynamics of proteins at work. *Curr. Opin. Biomed. Eng.* 12:8–17.
10. Schuler, B. 2013. Single-molecule FRET of protein structure and dynamics - a primer. *J. Nanobiotechnol.* 11:S2.
11. Lu, M., X. Ma, …, W. Mothes. 2019. Associating HIV-1 envelope glycoprotein structures with states on the virus observed by smFRET. *Nature.* 568:415–419.
12. Mooney, S. M., R. Qiu, …, K. R. Weninger. 2014. Cancer/testis antigen PAGE4, a regulator of c-Jun transactivation, is phosphorylated by homeodomain-interacting protein kinase 1, a component of the stress-response pathway. *Biochemistry.* 53:1670–1679.
13. Wozniak, A. K., G. F. Schröder, …, F. Oesterhelt. 2008. Single-molecule FRET measures bends and kinks in DNA. *Proc. Natl. Acad. Sci. USA.* 105:18337–18342.
14. Chung, H. S., J. M. Louis, and I. V. Gopich. 2016. Analysis of fluorescence lifetime and energy transfer efficiency in single-molecule photon trajectories of fast-folding proteins. *J. Phys. Chem. B.* 120:680–699.
15. Heo, W., K. Hasegawa, …, T. Tahara. 2022. Scanning two-dimensional fluorescence lifetime correlation spectroscopy: Conformational dynamics of DNA Holliday junction from microsecond to subsecond. *J. Phys. Chem. Lett.* 13:1249–1257.
16. Zeug, A., A. Woehler, …, E. G. Ponimaskin. 2012. Quantitative intensity-based fret approaches—a comparative snapshot. *Biophys. J.* 103:1821–1827.
17. Kuppa, S., J. Deveryshetty, …, E. Antony. 2022. Rtt105 regulates rpa function by configurationally stapling the flexible domains. *Nat. Commun.* 13:5152.
18. Kilic, Z., I. Sgouralis, and S. Pressé. 2021. Generalizing hmms to continuous time for fast kinetics: hidden markov jump processes. *Biophys. J.* 120:409–423.
19. Kapusta, P., M. Wahl, …, J. Enderlein. 2007. Fluorescence lifetime correlation spectroscopy. *J. Fluoresc.* 17:43–48.
20. Ishii, K., and T. Tahara. 2013. Two-dimensional fluorescence lifetime correlation spectroscopy. 1. principle. *J. Phys. Chem. B.* 117:11414–11422.
21. Otosu, T., K. Ishii, and T. Tahara. 2015. Microsecond protein dynamics observed at the single-molecule level. *Nat. Commun.* 6:7685.
22. Yoo, J., J.-Y. Kim, …, H. S. Chung. 2020. Fast three-color single-molecule FRET using statistical inference. *Nat. Commun.* 11:3336.
23. Lerner, E., A. Ingargiola, and S. Weiss. 2018. Characterizing highly dynamic conformational states: The transcription bubble in RNAP-promoter open complex as an example. *J. Chem. Phys.* 148:123315.
24. Saurabh, A., M. Safar, …, S. Pressé. 2022. Single photon smFRET. I. theory and conceptual basis. Preprint at bioRxiv. https://doi.org/10.1101/2022.07.20.500887.
25. Sgouralis, I., and S. Pressé. 2017. An introduction to infinite HMMs for single-molecule data analysis. *Biophys. J.* 112:2021–2029.
26. Sgouralis, I., S. Madaan, …, S. Pressé. 2019. A Bayesian nonparametric approach to single molecule Förster resonance energy transfer. *J. Phys. Chem. B.* 123:675–688.
27. Fox, E. B., E. B. Sudderth, …, A. S. Willsky. 2011. A sticky HDP-HMM with application to speaker diarization. *Ann. Appl. Stat.* 5:1020.
28. Teh, Y. W., M. I. Jordan, …, D. M. Blei. 2006. Hierarchical Dirichlet processes. *J. Am. Stat. Assoc.* 101:1566–1581.
29. Jayaram, S. 1994. A constructive definition of Dirichlet priors. *Stat. Sin.* 4:639.
30. Pitman, J. 2002. Poisson–Dirichlet and GEM invariant distributions for split-and-merge transformations of an interval partition. *Combinator. Probab. Comput.* 11:501–514.
31. Ferguson, T. S. 1973. A Bayesian analysis of some nonparametric problems. *Ann. Stat.* 1:209.
32. Gershman, S. J., and D. M. Blei. 2012. A tutorial on Bayesian nonparametric models. *J. Math. Psychol.* 56:1–12.
33. Sgouralis, I., M. Whitmore, …, S. Pressé. 2018. Single molecule force spectroscopy at high data acquisition: A Bayesian nonparametric analysis. *J. Chem. Phys.* 148:123320.
34. Tavakoli, M., J. N. Taylor, …, S. Pressé. 2017. Single molecule data analysis: An introduction. *Adv. Chem. Phys.* 162:205–305.
35. Tavakoli, M., S. Jazani, …, S. Pressé. 2020. Pitching single-focus confocal data analysis one photon at a time with Bayesian nonparametrics. *Phys. Rev. X.* 10:011021.
36. Tavakoli, M., S. Jazani, …, S. Pressé. 2020. Direct photon-by-photon analysis of time-resolved pulsed excitation data using Bayesian nonparametrics. *Cell Rep. Phys. Sci.* 1:100234.
37. Bryan, J. S., 4th, I. Sgouralis, and S. Pressé. 2022. Diffraction-limited molecular cluster quantification with Bayesian nonparametrics. *Nat. Comput. Sci.* 2:102–111.

38. Fazel, M., S. Jazani, …, S. Pressé. 2022. High resolution fluorescence lifetime maps from minimal photon counts. *ACS Photonics.* 9:1015–1025.
39. Okamoto, K., and Y. Sako. 2016. State transition analysis of spontaneous branch migration of the Holliday junction by photon-based single-molecule fluorescence resonance energy transfer. *Biophys. Chem.* 209:21–27.
40. Hohng, S., C. Joo, and T. Ha. 2004. Single-molecule three-color FRET. *Biophys. J.* 87:1328–1337.
41. McKinney, S. A., A. C. Déclais, …, T. Ha. 2003. Structural dynamics of individual Holliday junctions. *Nat. Struct. Biol.* 10:93–97.
42. McKinney, S. A., A. D. J. Freeman, …, T. Ha. 2005. Observing spontaneous branch migration of Holliday junctions one step at a time. *Proc. Natl. Acad. Sci. USA.* 102:5715–5720.
43. Panyutin, I. G., I. Biswas, and P. Hsieh. 1995. A pivotal role for the structure of the Holliday junction in DNA branch migration. *EMBO J.* 14:1819–1826.
44. Metropolis, N., A. W. Rosenbluth, …, E. Teller. 1953. Equation of state calculations by fast computing machines. *J. Chem. Phys.* 21:1087–1092.
45. Hastings, W. K. 1970. Monte Carlo sampling methods using Markov chains and their applications. *Biometrika.* 57:97–109.
46. Fazel, M., M. J. Wester, …, K. A. Lidke. 2019. Bayesian multiple emitter fitting using reversible jump Markov chain Monte Carlo. *Sci. Rep.* 9:13791.
47. Jazani, S., I. Sgouralis, …, S. Pressé. 2019. An alternative framework for fluorescence correlation spectroscopy. *Nat. Commun.* 10:3662.
48. Rabiner, L. R. 1989. A tutorial on hidden Markov models and selected applications in speech recognition. *Proc. IEEE.* 77:257–286.
49. Fazel, M., V. Alexander, …, S. Pressé. 2022. Fluorescence lifetime: beating the irf and interpulse window. Preprint at bioRxiv. https://doi.org/10.1101/2022.09.08.507224.
50. Gillespie, D. T. 1976. A general method for numerically simulating the stochastic time evolution of coupled chemical reactions. *J. Comput. Phys.* 22:403–434.
51. Kilic, Z., I. Sgouralis, …, S. Pressé. 2021. Extraction of rapid kinetics from smFRET measurements using integrative detectors. *Cell Rep. Phys. Sci.* 2:100409.
52. Karymov, M., D. Daniel, …, Y. L. Lyubchenko. 2005. Holliday junction dynamics and branch migration: single-molecule analysis. *Proc. Natl. Acad. Sci. USA.* 102:8186–8191.
53. Ferapontova, E. E., C. P. Mountford, …, A. R. Mount. 2008. Electrochemical control of a DNA Holliday Junction nanoswitch by $Mg2+$ ions. *Biosens. Bioelectron.* 24:422–428.
54. Pirchi, M., R. Tsukanov, …, E. Nir. 2016. Photon-by-photon hidden Markov model analysis for microsecond single-molecule FRET kinetics. *J. Phys. Chem. B.* 120:13065–13075.
55. Kilic, Z., I. Sgouralis, and S. Pressé. 2021. Generalizing HMMs to continuous time for fast kinetics: Hidden Markov jump processes. *Biophys. J.* 120:409–423.
56. Sebolt-Leopold, J. S., and J. M. English. 2006. Mechanisms of drug inhibition of signalling molecules. *Nature.* 441:457–462.
57. Jazani, S., I. Sgouralis, and S. Pressé. 2019. A method for single molecule tracking using a conventional single-focus confocal setup. *J. Chem. Phys.* 150, 114108.

Biophysical Reports, Volume *2*

Supplemental information

Single-photon smFRET. III. Application to pulsed illumination

Matthew Safar, Ayush Saurabh, Bidyut Sarkar, Mohamadreza Fazel, Kunihiko Ishii, Tahei Tahara, Ioannis Sgouralis, and Steve Pressé

Supplementary Information

Single Photon smFRET. III. Application to Pulsed Illumination

Matthew Safar[1,2], Ayush Saurabh[1], Bidyut Sarkar[3,4], Mohamadreza Fazel[1], Kunihiko Ishii[3,4], Tahei Tahara[3,4], Ioannis Sgouralis[5], and Steve Pressé[1,6]

[1]Center for Biological Physics, Department of Physics, Arizona State University, Tempe, AZ, USA
[2]Department of Mathematics and Statistical Science, Arizona State University, Tempe, AZ, USA
[3]Molecular Spectroscopy Laboratory, RIKEN, 2-1 Hirosawa, Wako, Saitama 351-0198, Japan
[4]Ultrafast Spectroscopy Research Team, RIKEN Center for Advanced Photonics (RAP), 2-1 Hirosawa, Wako, Saitama 351-0198, Japan
[5]Department of Mathematics, University of Tennessee Knoxville, Knoxville, TN, USA
[6]School of Molecular Sciences, Arizona State University, Phoenix, AZ, USA

Last updated: November 1, 2022

Contents

S1 Variables and Notation **S1**

S2 Likelihood for Pulsed Illumination **S3**
- S2.1 Excitation . . . S4
- S2.2 Photophysics . . . S5
- S2.3 Instrument Response Function . . . S7
- S2.4 Background . . . S8
  - S2.4.1 Laser background . . . S10
  - S2.4.2 Uniform background . . . S11

S3 Model Structure and Priors **S13**
S3.1 Parametric Model S13
S3.2 Nonparametric Model S14

S4 Sampling from the Posterior: Gibbs Algorithm Steps **S15**
S4.1 Photophysical rates S15
S4.2 Excitation Probability S15
S4.3 System State Trajectory S16
S4.4 Transition probabilities S17
S4.5 Base Distribution S17
S4.6 Optional Hyperparameters S18
S4.6.1 Transition probabilities concentration hyperparameter S18
S4.6.2 Sticky hyperparameter S18
S4.6.3 Base distribution concentration hyperparameter S19

S5 Estimation of pre-set Parameters **S20**
S5.1 IRF S20
S5.2 Background S20

S6 Parameters Used for Synthetic Data Generation **S22**

S7 Additional Parameter Estimates **S23**
S7.1 Synthetic Data with Two System States S23
S7.2 Synthetic Data with Three System States S24
S7.3 Experimental Data: 1 mM S25
S7.4 Experimental Data: 3 mM S26
S7.5 Experimental Data: 5 mM S27
S7.6 Experimental Data: 10 mM S28

Bibliography **S29**

S1 Variables and Notation

In this manuscript, we generally denote *vectors* and *collections* differently, even though mathematically they are very similar objects. Vectors, such as the probability vector $\boldsymbol{\pi}_m$ or the generator matrix $\mathbf{G}$ are bolded. On the other hand, collections, which represent groups of, in some sense, independent objects, such as the trajectory $s_{1:N}$ which is all states s_n grouped together are denoted with the colon notation $i : j$ to denote the range on indices from i to j.

Description	Variable	Units
The number of pulses	N	-
Length of pulse period	τ	ns
The macrotime of the n-th pulse	t_n	ns
The microtime of the n-th pulse	μ_n	ns or null
The measurement of the n-th pulse (μ_n^d, μ_n^a)	w_n	(ns,ns)
Number of states (weak limit in the nonparametric sense)	M	-
The m-th state	σ_m	-
The state at the nth pulse	s_n	-
The system state transition probability matrix	$\mathbf{\Pi}_\sigma$	-
The m-th row of $\mathbf{\Pi}_\sigma$	$\boldsymbol{\pi}_m$	-
Initial state probability vector	$\boldsymbol{\pi}_0$	-
Concentration hyperparameter for $\pi_{1:M}$ and π_0	α	-
Base distribution over states in the iHMM	$\boldsymbol{\beta}$	-
Concentration hyperparameter for β	γ	-
Donor relaxation rate	λ_d	ns^{-1}
Acceptor relaxation rate	λ_a	ns^{-1}
FRET rate of m-th state	λ_{FRET}^m	ns^{-1}
Probability of donor becoming excited by a pulse	π_{ex}	-
Excitation event at time n	a_n	-
Direct acceptor excitation coefficient	k_a	-
Efficiency of the donor channel	η_d	-
Efficiency of the acceptor channel	η_a	-
Probability of no detector leakage in donor channel	ϕ_{dd}	-
Probability of no detector leakage in acceptor channel	ϕ_{aa}	-
Probability of donor channel laser background photon	p_{bd}	-
Probability of acceptor channel laser background photon	p_{ba}	-
Probability of donor channel uniform background photon	p_{dd}	-
Probability of acceptor channel laser background photon	p_{da}	-
Donor channel IRF delay mean	μ_d^{IRF}	ns
Donor channel IRF delay variance	ν_d^{IRF}	ns^2
Acceptor channel IRF delay mean	μ_a^{IRF}	ns
Acceptor channel IRF delay variance	ν_a^{IRF}	ns^2
The collection of all learned parameters (shorthand)	ϑ	-

Table S1: **Table of Variables and Units.** For the convenience of the readers, we include a table with the quantities discussed in this paper and their corresponding symbols.

S2 Likelihood for Pulsed Illumination

In order to perform inference over the parameters as described in the main text [1], we use the likelihood described in Eq. 6 of the main text [1]

$$L = p(\boldsymbol{w}|\boldsymbol{\rho}_{start}, \mathbf{\Pi}_\sigma, \mathbf{G}_\psi) \propto \boldsymbol{\rho}_{start}\mathbf{\Pi}_1^\sigma \mathbf{\Pi}_2^\sigma \ldots \mathbf{\Pi}_N^\sigma \boldsymbol{\rho}_{norm}^T, \tag{S1}$$

where $\boldsymbol{\rho}_{start}$ is a vector collecting all the initial probabilities. $\mathbf{G}_\psi$ is the photophysical generator matrix given in Eq. 4 of the first companion manuscript [2]. Moreover, $\mathbf{\Pi}_n^\sigma$ is the reduced system state propagator for the n-th interpulse period given by

$$\mathbf{\Pi}_n^\sigma = \mathbf{\Pi}_\sigma \odot \mathbf{D}_n^\sigma, \tag{S2}$$

where $\odot$ denotes element-by-element product. Here, $\mathbf{D}_n^\sigma$ is the detection matrix with elements

$$(\mathbf{D}_n^\sigma)_{s_n \to \sigma_j} = p(w_n|s_n, \mathbf{G}_\psi) = \boldsymbol{\rho}_{ground}\mathbf{Q}_n^\psi(s_n)\boldsymbol{\rho}_{norm}^T, \tag{S3}$$

as described in Sec. 3 of the main text [1]. Here, $\boldsymbol{\rho}_{ground}$ is the probability vector where both donor and acceptor are in the ground state. Futhermore, $\mathbf{Q}_n^\psi(s_n)$ is the photophysical propagator for n-th interpulse period.

The photophysical propagators take different forms depending on the observation during an interpulse period. To derive the explicit forms of these photophysical propagators, we start from the explicit from of the photophysical generator matrix $\mathbf{G}_\psi$ for a given system state, s_n, as

$$\mathbf{G}_\psi = \begin{pmatrix} * & \lambda_{ex}(t) & \lambda_{direct}(t) \\ \lambda_d & * & \lambda_{s_n}^{FRET} \\ \lambda_a & 0 & * \end{pmatrix}, \tag{S4}$$

where λ_{ex}, λ_{direct}, λ_d, λ_a and $\lambda_{s_n}^{FRET}$, respectively, denote donor excitation, direct acceptor excitation, donor relaxation, acceptor relaxation and FRET rates. As such, the propagators for empty and nonempty pulses are obtained by replacing the photophysical generator matrix in the generic propagators described in Sec. 2.5.1 of the first companion manuscript [2]

$$\mathbf{Q}_n^\psi = \mathbf{exp}\left(\int_0^{\delta_{pulse}} d\delta \mathbf{G}_\psi^{non}(\delta)\right) \mathbf{exp}\Big((\tau - \delta_{pulse})\, \mathbf{G}_\psi^{dark}\Big), \tag{S5}$$

$$\begin{aligned}\mathbf{Q}_n^\psi = \mathbf{exp}\left(\int_0^{\delta_{pulse}} d\delta \mathbf{G}_\psi^{non}(\delta)\right) \Bigg(\int_0^{\delta_{IRF}} d\epsilon_n \, \mathbf{exp}\left[(\mu_n - \delta_{pulse} - \epsilon_n)\, \mathbf{G}_\psi^{dark}\right] \mathbf{G}_\psi^{rad} \\ \times \mathbf{exp}\left[(\tau - \mu_n + \epsilon_n)\, \mathbf{G}_\psi^{dark}\right] f(\epsilon_n)\Bigg),\end{aligned} \tag{S6}$$

for empty and nonempty pulses, respectively. The different generator matrices above are the reduced forms of $\mathbf{G}^{non}$, $\mathbf{G}^{dark}$ and $\mathbf{G}^{rad}$ introduced in the first companion manuscript [2] Sec. 2.3, now containing only photophysical transitions. In what follows, we will derive these reduced generator matrices and calculate different terms involved in the likelihoods above. We then proceed to take into account the background and instrument response function (IRF) in the likelihoods.

S2.1 Excitation

To construct the likelihood for a pulse, we begin by considering the laser pulse itself where we expect no transition other than fluorophore excitation occurring during this period. This assumption is reasonable since pulse duration is too short (often of the order of 100 ps) compared to fluorophore lifetimes. Therefore, the generator matrix for this period is derived from Eq. S4 by setting $\lambda_d = \lambda_a = \lambda_{FRET} = 0$, leading to

$$\mathbf{G}_{\psi}^{non} = \begin{bmatrix} * & \lambda_{ex}(t) & \lambda_{direct}(t) \\ 0 & 0 & 0 \\ 0 & 0 & 0 \end{bmatrix}. \tag{S7}$$

Therefore, the first term in propagators Eq. S5-S6 is obtained as

$$\mathbf{\Pi}_{\psi}^{pulse} = \mathbf{exp}\left(\int_0^{\delta_{pulse}} \begin{bmatrix} * & \lambda_{ex}(t) & \lambda_{direct}(t) \\ 0 & 0 & 0 \\ 0 & 0 & 0 \end{bmatrix} dt \right), \tag{S8}$$

where $\mathbf{\Pi}_{\psi}^{non}$ represents the nonradiative propagator matrix during the laser pulse.

The above expression can be further simplified by taking into account the fact that both excitation rates are proportional to the pulse intensity with different constants of proportionality [3]. Consequently, we can write $\lambda_{direct} = k_a \lambda_{ex}$, where k_a is the ratio of the proportionality constants. The resulting propagator is thus

$$\mathbf{\Pi}_{\psi}^{pulse} = \mathbf{exp}\left(\int_0^{\delta_{pulse}} \begin{bmatrix} * & \lambda_{ex}(t) & k_a\lambda_{ex}(t) \\ 0 & 0 & 0 \\ 0 & 0 & 0 \end{bmatrix} dt \right). \tag{S9}$$

This integral and the subsequent matrix exponential can be solved analytically, with the result

$$\mathbf{\Pi}_{\psi}^{pulse} = \begin{pmatrix} 1 - \pi_{ex} - k_a\pi_{ex} & \pi_{ex} & k_a\pi_{ex} \\ 0 & 0 & 0 \\ 0 & 0 & 0 \end{pmatrix}, \tag{S10}$$

where

$$\pi_{ex} = \frac{1}{1+k_a}\left(1 - \mathbf{exp}\left(-(1+k_a)\int_0^{\delta_{pulse}} \lambda_{ex}(t)dt\right)\right), \tag{S11}$$

where π_{ex} and $k_a\pi_{ex}$ are the probabilities that the donor or acceptor is directly excited by the pulse, respectively. This quantity is the same for all the pulses because the molecule is immobilized.

Now, using the obtained propagator for the pulse we can find the photophysical state probability vector immediately after the pulse $\boldsymbol{\rho}_{pulse}$. It is given by

$$\boldsymbol{\rho}_{pulse} = \boldsymbol{\rho}_{ground}\mathbf{\Pi}_{\psi}^{pulse} = \begin{pmatrix} 1 - \pi_{ex} - k_a\pi_{ex}, & \pi_{ex}, & k_a\pi_{ex} \end{pmatrix}, \tag{S12}$$

where $\boldsymbol{\rho}_{ground}$ is the photophysical state probability vector at the beginning of the pulse when both fluorophores are in the ground state by assumption (2) in Sec. 3 of the main text [1].

S2.2 Photophysics

Next, we compute the remaining terms in propagators of Eqs. S5-S6. To do so, we first calculate the generator matrices in those terms, namely, $\mathbf{G}_{\psi}^{dark}$ for no photon detection, and $\mathbf{G}_{\psi}^{rad}$ for photon detection. These two generators describe events after the laser pulse and before the next laser pulse where no fluorophore excitation may take place and thus we have $\lambda_{ex} = \lambda_{direct} = 0$.

Now, for an empty interpulse period where there is no photon detection, there is still a chance for emitted photons that are not detected quantified by detector efficiencies η_d and η_a for donor and acceptor channels, respectively. Therefore, we can write (see Sec. 2.5.1 in the first companion manuscript [2])

$$\mathbf{G}_{\psi}^{dark} = \begin{pmatrix} 0 & 0 & 0 \\ (1-\eta_d)\lambda_d & -\lambda_d - \lambda_{s_n}^{FRET} & \lambda_{s_n}^{FRET} \\ (1-\eta_a)\lambda_a & 0 & -\lambda_a \end{pmatrix}. \tag{S13}$$

For nonempty interpulse periods, only radiative transitions associated with the detected photon are possible at that detection moment, therefore we further set the nonradiative transition rates $\lambda_{FRET} = 0$. If a photon is detected in the donor channel, the radiative propagator is thus

$$\mathbf{G}_{\psi}^{rad(D)} = \begin{pmatrix} 0 & 0 & 0 \\ \eta_d \phi_{dd} \lambda_d & 0 & 0 \\ \eta_d (1-\phi_{aa})\lambda_a & 0 & 0 \end{pmatrix}, \tag{S14}$$

and for the acceptor channel

$$\mathbf{G}_{\psi}^{rad(A)} = \begin{pmatrix} 0 & 0 & 0 \\ \eta_a (1-\phi_{dd})\lambda_d & 0 & 0 \\ \eta_a \phi_{aa} \lambda_a & 0 & 0 \end{pmatrix}, \tag{S15}$$

where $(1-\phi_{dd})$ and $(1-\phi_{aa})$ denote the crosstalk probabilities for donor and acceptor channels, respectively.

Now, if we ignore the background and the IRF for the moment, using the obtained generators above, the elements of the detection matrix $(\mathbf{D}_n^{\sigma})_{s_n \to \sigma_j}$ for an empty pulse, a nonempty pulse with a donor photon, and a nonempty pulses with an acceptor photon are, respectively, given as

$$(\mathbf{D}_n^{\sigma})_{s_n \to \sigma_j}(\emptyset, \emptyset) = \boldsymbol{\rho}_{pulse}\, \mathbf{exp}(\tau \mathbf{G}_{\psi}^{dark}) \boldsymbol{\rho}_{norm}^{T}, \quad \text{no photon,} \tag{S16}$$

$$(\mathbf{D}_n^{\sigma})_{s_n \to \sigma_j}(\mu, \emptyset) = \boldsymbol{\rho}_{pulse}\, \mathbf{exp}(\mu_n \mathbf{G}_{\psi}^{dark}) \mathbf{G}_{\psi}^{rad(D)}\, \mathbf{exp}((\tau - \mu_n)\, \mathbf{G}_{\psi}^{dark}) \boldsymbol{\rho}_{norm}^{T}, \quad \text{donor photon} \tag{S17}$$

$$(\mathbf{D}_n^{\sigma})_{s_n \to \sigma_j}(\emptyset, \mu) = \boldsymbol{\rho}_{pulse}\, \mathbf{exp}(\mu_n \mathbf{G}_{\psi}^{dark}) \mathbf{G}_{\psi}^{rad(A)}\, \mathbf{exp}((\tau - \mu_n)\, \mathbf{G}_{\psi}^{dark}) \boldsymbol{\rho}_{norm}^{T}, \quad \text{acceptor photon} \tag{S18}$$

where we ignored the integrals due to IRF in Eq. S5-S6. Moreover, $\emptyset$ and μ as the first input, respectively denote no photon and a photon with arrival time μ from the donor

channel. The same applies to the second input but for the acceptor channel. These elements can be analytically solved as

$$
\begin{aligned}
&(\mathbf{D}_n^{\sigma})_{s_n\to\sigma_j}(\emptyset,\emptyset) = \\
&\boldsymbol{\rho}_{pulse}\begin{pmatrix} 1 & 0 & 0 \\ A(\tau) & \mathbf{exp}(-(\lambda_d+\lambda_{s_n}^{FRET})\tau) & B(\tau) \\ (1-\eta_a)(1-\mathbf{exp}(-\lambda_a\tau)) & 0 & \mathbf{exp}(-\lambda_a\tau)\end{pmatrix}\boldsymbol{\rho}_{norm}^T, \qquad \text{(S19)}\\
&(\mathbf{D}_n^{\sigma})_{s_n\to\sigma_j}(\mu,\emptyset) = \\
&\boldsymbol{\rho}_{pulse}\begin{pmatrix} 0 & 0 & 0 \\ \eta_d\phi_{dd}\lambda_d\,\mathbf{exp}(-(\lambda_d+\lambda_{s_n}^{FRET})\mu)+\eta_d(1-\phi_{aa})\lambda_a B(\mu) & 0 & 0 \\ \eta_d(1-\phi_{aa})\lambda_a\,\mathbf{exp}(-\lambda_a\mu) & 0 & 0\end{pmatrix}\boldsymbol{\rho}_{norm}^T, \qquad \text{(S20)}\\
&(\mathbf{D}_n^{\sigma})_{s_n\to\sigma_j}(\emptyset,\mu) = \\
&\boldsymbol{\rho}_{pulse}\begin{pmatrix} 0 & 0 & 0 \\ \eta_a(1-\phi_{dd})\lambda_d\,\mathbf{exp}(-(\lambda_d+\lambda_{s_n}^{FRET})\mu)+\eta_a\phi_{aa}\lambda_a B(\mu) & 0 & 0 \\ \eta_a\phi_{aa}\lambda_a\,\mathbf{exp}(-\lambda_a\mu) & 0 & 0\end{pmatrix}\boldsymbol{\rho}_{norm}^T, \qquad \text{(S21)}
\end{aligned}
$$

where τ is the interpulse period and

$$
A(t) = \frac{(1-\eta_d)\lambda_d+(1-\eta_a)\lambda_{s_n}^{FRET}}{\lambda_d+\lambda_{s_n}^{FRET}}(1-\mathbf{exp}(-(\lambda_d+\lambda_{s_n}^{FRET})t)), \qquad \text{(S22)}
$$

$$
B(t) = \frac{\lambda_{s_n}^{FRET}}{-\lambda_d-\lambda_{s_n}^{FRET}+\lambda_a}(\mathbf{exp}(-(\lambda_d+\lambda_{s_n}^{FRET})t)-\mathbf{exp}(-\lambda_a t)). \qquad \text{(S23)}
$$

These can be further simplified by making the assumption that interpulses period is long in comparison to the fluorophore lifetimes (assumption (2) above). In essence, we take $\tau\to\infty$. Therefore, the elements of the detection matrix when no photon is detected becomes

$$
\lim_{\tau\to\infty}(\mathbf{D}_n^{\sigma})_{s_n\to\sigma_j}(\emptyset,\emptyset) = \boldsymbol{\rho}_{pulse}\begin{pmatrix} 1 & 0 & 0 \\ \frac{(1-\eta_d)\lambda_d+(1-\eta_a)\lambda_{s_n}^{FRET}}{\lambda_d+\lambda_{s_n}^{FRET}} & 0 & 0 \\ (1-\eta_a) & 0 & 0\end{pmatrix}\boldsymbol{\rho}_{norm}^T. \qquad \text{(S24)}
$$

Now, since $\boldsymbol{\rho}_{norm} = [1,\ 1,\ 1]$, these matrices can be reduced to vectors by incorporating $\boldsymbol{\rho}_{norm}^T$ as

$$
(\mathbf{D}_n^{\sigma})_{s_n\to\sigma_j}(\emptyset,\emptyset) = \boldsymbol{\rho}_{pulse}\begin{pmatrix} 1 \\ \frac{(1-\eta_d)\lambda_d+(1-\eta_a)\lambda_{s_n}^{FRET}}{\lambda_d+\lambda_{s_n}^{FRET}} \\ (1-\eta_a)\end{pmatrix}, \qquad \text{(S25)}
$$

$$
(\mathbf{D}_n^{\sigma})_{s_n\to\sigma_j}(\mu,\emptyset) = \boldsymbol{\rho}_{pulse}\begin{pmatrix} 0 \\ \eta_d\phi_{dd}\lambda_d\,\mathbf{exp}(-(\lambda_d+\lambda_{s_n}^{FRET})\mu)+\eta_d(1-\phi_{aa})\lambda_a B(\mu) \\ \eta_d(1-\phi_{aa})\lambda_a\,\mathbf{exp}(-\lambda_a\mu)\end{pmatrix}, \qquad \text{(S26)}
$$

$$
(\mathbf{D}_n^{\sigma})_{s_n\to\sigma_j}(\emptyset,\mu) = \boldsymbol{\rho}_{pulse}\begin{pmatrix} 0 \\ \eta_a(1-\phi_{dd})\lambda_d\,\mathbf{exp}(-(\lambda_d+\lambda_{s_n}^{FRET})\mu)+\eta_a\phi_{aa}\lambda_a B(\mu) \\ \eta_a\phi_{aa}\lambda_a\,\mathbf{exp}(-\lambda_a\mu)\end{pmatrix}. \qquad \text{(S27)}
$$

Additionally, it is sometimes convenient to consider the likelihood of only detecting a donor or acceptor photon, regardless of the photon arrival time. We find this by marginalizing over the arrival times, and denote these marginalized likelihoods by

$$(\hat{\mathbf{D}}_n^{\sigma})_{s_n\to\sigma_j}(d) = \int_0^{\infty} (\mathbf{D}_n^{\sigma})_{s_n\to\sigma_j}(t,\emptyset)dt = \boldsymbol{\rho}_{pulse}\begin{pmatrix} 0 \\ \eta_d\phi_{dd}(1-\varepsilon_{s_n}^{FRET}) + \eta_d(1-\phi_{aa})\varepsilon_{s_n}^{FRET} \\ \eta_d(1-\phi_{aa}) \end{pmatrix}, \tag{S28}$$

$$(\hat{\mathbf{D}}_n^{\sigma})_{s_n\to\sigma_j}(a) = \int_0^{\infty} (\mathbf{D}_n^{\sigma})_{s_n\to\sigma_j}(\emptyset,t)dt = \boldsymbol{\rho}_{pulse}\begin{pmatrix} 0 \\ \eta_d(1-\phi_{dd})(1-\varepsilon_{s_n}^{FRET}) + \eta_d\phi_{aa}\varepsilon_{s_n}^{FRET} \\ \eta_a\phi_{aa} \end{pmatrix}, \tag{S29}$$

where $\varepsilon_{s_n}^{FRET} = \lambda_{s_n}^{FRET}/(\lambda_d + \lambda_{FRET}^{s_n})$ is the FRET efficiency for the system state s_n. Here, $\hat{\mathbf{D}}_n^{\sigma}$ denotes marginalization over arrival times.

In what follows, we will describe how to include the IRF and background into the derived detection matrices in this section.

S2.3 Instrument Response Function

The IRF refers to the delay between a photon arrival to a detector and the arrival time reported by the detector due to the electronics. We incorporate it by concluding that the reported arrival time t_{rep} is the sum of two random variables, t_{arrive} and t_{IRF}, as follows

$$t_{rep} = t_{arrive} + t_{IRF}. \tag{S30}$$

As it is a sum of two random variables the resulting distribution of t_{rep} is a convolution of the photon arrival time distribution with the IRF distribution. Here, we assume that the IRF is distributed according to

$$t_{IRF} \sim \textbf{Normal}(\mu_{IRF}, \nu_{IRF}), \tag{S31}$$

with each channel having a unique mean μ_{IRF} and variance ν_{IRF}. Moreover, the distribution of t_{arrive} is described by $(\mathbf{D}_n^{\sigma})_{s_n\to\sigma_j}$ derived in the previous section.

Now, we can obtain the likelihood in the presence of the IRF by calculating the convolution implied by Eq. S30. That is obtained as follows

$$(\mathbf{D}_n^{\sigma})_{s_n\to\sigma_j}^{IRF}(\mu,\emptyset) = \boldsymbol{\rho}_{pulse}\begin{pmatrix} 0 \\ \eta_d\phi_{dd}\lambda_d f_d(\mu,\lambda_d+\lambda_{s_n}^{FRET}) + \eta_d(1-\phi_{aa})\lambda_a B_{f_d}(\mu) \\ \eta_d(1-\phi_{aa})\lambda_a f_d(\mu,\lambda_a) \end{pmatrix}, \tag{S32}$$

$$(\mathbf{D}_n^{\sigma})_{s_n\to\sigma_j}^{IRF}(\emptyset,\mu) = \boldsymbol{\rho}_{pulse}\begin{pmatrix} 0 \\ \eta_a(1-\phi_{dd})\lambda_d f_a(\mu,\lambda_d+\lambda_{s_n}^{FRET}) + \eta_a\phi_{aa}\lambda_a B_{f_a}(\mu) \\ \eta_a\phi_{aa}\lambda_a f_a(\mu,\lambda_a) \end{pmatrix}, \tag{S33}$$

where

$$B_{f_d}(t) = \frac{\lambda_{s_n}^{FRET}}{-\lambda_d - \lambda_{s_n}^{FRET} + \lambda_a}(f_d(t, \lambda_d + \lambda_{s_n}^{FRET}) - f_d(t, \lambda_a)), \tag{S34}$$

$$f_d(t, \lambda) = \frac{1}{2}\mathbf{exp}\left(\frac{\lambda}{2}(2\mu_d^{IRF} + \lambda\nu_d^{IRF} - 2t)\right)\mathbf{erfc}\left(\frac{\mu_d^{IRF} + \lambda\nu_d^{IRF} - t}{\sqrt{2\nu_d^{IRF}}}\right), \tag{S35}$$

$$B_{f_a}(t) = \frac{\lambda_{s_n}^{FRET}}{-\lambda_d - \lambda_{s_n}^{FRET} + \lambda_a}(f_a(t, \lambda_d + \lambda_{s_n}^{FRET}) - f_a(t, \lambda_a)), \tag{S36}$$

$$f_a(t, \lambda) = \frac{1}{2}\mathbf{exp}\left(\frac{\lambda}{2}(2\mu_a^{IRF} + \lambda\nu_a^{IRF} - 2t)\right)\mathbf{erfc}\left(\frac{\mu_a^{IRF} + \lambda\nu_a^{IRF} - t}{\sqrt{2\nu_a^{IRF}}}\right), \tag{S37}$$

where $\mathbf{erfc}(\cdot) = 1 - \mathbf{erf}(\cdot)$ is the *complementary error function.* Moreover, note that $(\mathbf{D}_n^{\sigma})_{s_n \to \sigma_j}^{IRF}(\emptyset, \emptyset) = (\mathbf{D}_n^{\sigma})_{s_n \to \sigma_j}(\emptyset, \emptyset)$ since there is no photon and thus no IRF effect.

S2.4 Background

In this section, we proceed to include background emissions in our formulation following Sec. 2.6 of the first comapnion manuscript [2]. The background photons come from extra light sources present in the environment in addition to the FRET pair. Such source of photon is, in general, characterized by two components: 1) photon emission probabilities for each channel; and 2) distribution of photon arrival times over the interpulse window.

Here, we first assume p_d and p_a to be the probability that a photon is emitted in the donor and acceptor channels, respectively. Further, let $g_d(t)$ and $g_a(t)$ be probability density functions that describe the distribution of background photons' arrival times within the interpulse window for the donor and acceptor channels, respectively. Moreover, note that if the source is such that there is some relationship between donor and acceptor photons, we would additionally require a joint probability distribution $g_{da}(t_d, t_a)$, but in the case of background, we assume that the channels are independent. Therefore, the distribution over measurements for this source is

$$p_{bg}(w_n) = \begin{cases} (1-p_d)(1-p_a) & w_n = (\emptyset, \emptyset) \\ p_d g_d(\mu_d)(1-p_a) & w_n = (\mu_d, \emptyset) \\ (1-p_d)p_a g_a(\mu_a) & w_n = (\emptyset, \mu_a) \\ p_{bd}p_{ba}g_d(\mu_d)g_a(\mu_a) & w_n = (\mu_d, \mu_a). \end{cases} \tag{S38}$$

In the presence of a background source, we run into the complication that, in most single photon pulsed illumination setups, only the first photon arriving to a detector channel is recorded. This means that there is a competition between photons from different sources, namely, donor fluorophore, acceptor fluorophore, and background, to first reaching the detector. In many cases, this effect can be ignored, but here we take it into account for generality. In this case, we can write the likelihood in the presence of background but absence of the

IRF, $(\mathbf{D}_n^\sigma)^{bg}_{s_n\to\sigma_j}$, as follows

$$(\mathbf{D}_n^\sigma)^{bg}_{s_n\to\sigma_j}(w_n) =$$
$$\begin{cases} p_{bg}(\emptyset,\emptyset)(\mathbf{D}_n^\sigma)_{s_n\to\sigma_j}(\emptyset,\emptyset) & w_n=(\emptyset,\emptyset) \\ p_{bg}(\emptyset,\emptyset)(\mathbf{D}_n^\sigma)_{s_n\to\sigma_j}(\mu_d,\emptyset)+p_{bg}(\mu_d,\emptyset)(\mathbf{D}_n^\sigma)_{s_n\to\sigma_j}(\emptyset,\emptyset)+m_d(\mu_d,\emptyset) & w_n=(\mu_d,\emptyset) \\ p_{bg}(\emptyset,\emptyset)(\mathbf{D}_n^\sigma)_{s_n\to\sigma_j}(\emptyset,\mu_a)+p_{bg}(\emptyset,\mu_a)(\mathbf{D}_n^\sigma)_{s_n\to\sigma_j}(\emptyset,\emptyset)+m_a(\emptyset,\mu_a) & w_n=(\emptyset,\mu_a) \\ p_{bg}(\emptyset,\mu_a)(\mathbf{D}_n^\sigma)_{s_n\to\sigma_j}(\mu_d,\emptyset)+p_{bg}(\mu_d,\emptyset)(\mathbf{D}_n^\sigma)_{s_n\to\sigma_j}(\emptyset,\mu_a) & w_n=(\mu_d,\mu_a) \\ +p_{bg}(\mu_d,\mu_a)(\mathbf{D}_n^\sigma)_{s_n\to\sigma_j}(\emptyset,\emptyset)+M_d(\mu_d,\mu_a)+M_a(\mu_d,\mu_a), & \end{cases} \tag{S39}$$

where p_{bg} is given by Eq. S38 and $(\mathbf{D}_n^\sigma)_{s_n\to\sigma_j}$ is the likelihood for signal photons, *i.e.*, photons from fluorophores. Further, m_d, m_a, M_d and M_a correspond to the cases where both background and signal photon are present, but only the smaller arrival time is detected. These are derived by finding the distribution of the minimum arrival times between the competing photons as follows

$$\begin{aligned} m_d(t,\emptyset) =& p_d(1-p_a)\Bigg[(g_d(t)(\hat{\mathbf{D}}_n^\sigma)_{s_n\to\sigma_j}(d)+(\mathbf{D}_n^\sigma)_{s_n\to\sigma_j}(t,\emptyset)-g_d(t)\left(\int_0^t(\mathbf{D}_n^\sigma)_{s_n\to\sigma_j}(s,\emptyset)ds\right) \\ &-\left(\int_0^t g_d(s)ds\right)(\mathbf{D}_n^\sigma)_{s_n\to\sigma_j}(t,\emptyset)\Bigg], \end{aligned} \tag{S40}$$

$$\begin{aligned} m_a(\emptyset,t) =& (1-p_d)p_a\Bigg[g_a(t)(\hat{\mathbf{D}}_n^\sigma)_{s_n\to\sigma_j}(a)+(\mathbf{D}_n^\sigma)_{s_n\to\sigma_j}(t,\emptyset)-g_a(t)\left(\int_0^t(\mathbf{D}_n^\sigma)_{s_n\to\sigma_j}(s,\emptyset)ds\right) \\ &-\left(\int_0^t g_a(s)ds\right)(\mathbf{D}_n^\sigma)_{s_n\to\sigma_j}(t,\emptyset)\Bigg], \end{aligned} \tag{S41}$$

$$\begin{aligned} M_d(t_d,t_a) =& p_dp_a\Bigg[g_d(t_d)(\hat{\mathbf{D}}_n^\sigma)_{s_n\to\sigma_j}(d)+(\mathbf{D}_n^\sigma)_{s_n\to\sigma_j}(t_d,\emptyset)-g_d(t)\left(\int_0^{t_d}(\mathbf{D}_n^\sigma)_{s_n\to\sigma_j}(s,\emptyset)ds\right) \\ &-\left(\int_0^{t_d} g_d(s)ds\right)(\mathbf{D}_n^\sigma)_{s_n\to\sigma_j}(t_d,\emptyset)\Bigg]g_a(t_a), \end{aligned} \tag{S42}$$

$$\begin{aligned} M_a(t_d,t_a) =& p_dp_ag_d(t_d)\Bigg[g_a(t_a)(\hat{\mathbf{D}}_n^\sigma)_{s_n\to\sigma_j}(a)+(\mathbf{D}_n^\sigma)_{s_n\to\sigma_j}(t_a,\emptyset)-g_a(t_a)\left(\int_0^{t_a}(\mathbf{D}_n^\sigma)_{s_n\to\sigma_j}(s,\emptyset)ds\right) \\ &-\left(\int_0^{t_a} g_a(s)ds\right)(\mathbf{D}_n^\sigma)_{s_n\to\sigma_j}(t_a,\emptyset)\Bigg], \end{aligned} \tag{S43}$$

where $(\hat{\mathbf{D}}_n^\sigma)_{s_n\to\sigma_j}$ is the marginalized element introduced in Eq. S28.

Using this general method of incorporating additional light sources into our framework, we can add the two most prominant background sources observed in the data: 1) laser photons which are distributed the same as laser pulse and termed laser background; and 2) uniform background which are uniformly distributed over the interpulse window and termed uniform background. In what follows, we will discuss the inclusion of these two backgrounds in our model.

S2.4.1 Laser background

The primary goal of this section is constructing a general method for adding background photons originating from the laser source to our pulsed illumination framework. These photons arrive to the detector distributed according to the intensity of the laser pulse across the interpulse window. Moreover, since the pulse width is extremely narrow, it can be effectively considered as a delta function. Therefore, using our description for a generic background source (Eq. S38), we can describe this laser background as

$$p_{bg}(w_n) = \begin{cases} (1-p_{bd})(1-p_{ba}) & w_n = (\emptyset,\emptyset) \\ p_{bd}\delta(\mu_d)(1-p_{ba}) & w_n = (\mu_d,\emptyset) \\ (1-p_{bd})p_{ba}\delta(\mu_a) & w_n = (\emptyset,\mu_a) \\ p_{bd}p_{ba}\delta(\mu_d)\delta(\mu_a) & w_n = (\mu_d,\mu_a) \end{cases}, \tag{S44}$$

where we used $g_{d\backslash a}(\mu_{d\backslash a}) = \delta(\mu_{d\backslash a})$ for laser photons.

Since the laser photons arrive exactly at the beginning of the interpulse window, they are going to naturally win the competition between multiple present photons from different sources. This in turn simplifies the terms m_d, m_a, M_d, and M_a in Eq. S40-S43 for laser photons as follows

$$m^l_d(\mu_d,\emptyset) = p_{bd}(1-p_{ba})(\hat{\mathbf{D}}^\sigma_n)_{s_n\to\sigma_j}(d)\delta(\mu_d), \tag{S45}$$
$$m^l_a(\emptyset,\mu_a) = (1-p_{bd})p_{ba}(\hat{\mathbf{D}}^\sigma_n)_{s_n\to\sigma_j}(a)\delta(\mu_a), \tag{S46}$$
$$M^l_d(\mu_d,\mu_a) = p_{bd}p_{ba}(\hat{\mathbf{D}}^\sigma_n)_{s_n\to\sigma_j}(d)\delta(\mu_d)\delta(\mu_a), \tag{S47}$$
$$M^l_a(\mu_d,\mu_a) = p_{bd}p_{ba}(\hat{\mathbf{D}}^\sigma_n)_{s_n\to\sigma_j}(a)\delta(\mu_d)\delta(\mu_a). \tag{S48}$$

Here, the marginalized terms $(\hat{\mathbf{D}}^\sigma_n)_{s_n\to\sigma_j}$ (defined in Eq. S28) account for the probability of receiving a signal photon from the fluorophore, even if it is not detected due to the laser background photon arriving first.

Now, by substituting Eq. S44-S48 in Eq. S39 we can derive the likelihood model of the photons reaching to the detector in the presence of laser photons. To derive the reported arrival time likelihood model, we still need to add the IRF effect. To do so, we need to convolve the IRF with the delta function that describes the laser photon distributions across the interpulse window. This results in the IRF itself which is given by a Normal distribution

$$\int d\omega\delta(t-\omega)\mathbf{Normal}(\omega;\mu_{IRF},\nu_{IRF}) = \mathbf{Normal}(t;\mu_{IRF},\nu_{IRF}), \tag{S49}$$

where ω is an auxiliary variable. As such, using the $(\mathbf{D}^\sigma_n)^{IRF}_{s_n\to\sigma_j}$ in Eqs. S32-S33 and the background terms as described above, we obtain the likelihood model in the presence of

laser background $(\mathbf{D}_n^\sigma)^{laser}_{s_n\to\sigma_j}$ as

$$(\mathbf{D}_n^\sigma)^{laser}_{s_n\to\sigma_j}(\emptyset,\emptyset) = (1-p_{bd})(1-p_{ba})(\mathbf{D}_n^\sigma)_{s_n\to\sigma_j}(\emptyset,\emptyset), \tag{S50}$$

$$\begin{aligned}(\mathbf{D}_n^\sigma)^{laser}_{s_n\to\sigma_j}(\mu_d,\emptyset) =& (1-p_{bd})(1-p_{ba})(\mathbf{D}_n^\sigma)^{IRF}_{s_n\to\sigma_j}(\mu_d,\emptyset)\\ &+ p_{bd}\mathbf{Normal}(\mu_d;\mu_d^{IRF},\nu_d^{IRF})(1-p_{ba})(\mathbf{D}_n^\sigma)_{s_n\to\sigma_j}(\emptyset,\emptyset)\\ &+ p_{bd}(1-p_{ba})(\hat{\mathbf{D}}_n^\sigma)_{s_n\to\sigma_j}(d)\mathbf{Normal}(\mu_d;\mu_d^{IRF},\nu_d^{IRF}),\end{aligned} \tag{S51}$$

$$\begin{aligned}(\mathbf{D}_n^\sigma)^{laser}_{s_n\to\sigma_j}(\emptyset,\mu_a) =& (1-p_{bd})(1-p_{ba})(\mathbf{D}_n^\sigma)^{IRF}_{s_n\to\sigma_j}(\emptyset,\mu_a)\\ &+ (1-p_{bd})p_{ba}\mathbf{Normal}(\mu_a;\mu_a^{IRF},\nu_a^{IRF})(\mathbf{D}_n^\sigma)_{s_n\to\sigma_j}(\emptyset,\emptyset)\\ &+ (1-p_{bd})p_{ba}(\hat{\mathbf{D}}_n^\sigma)_{s_n\to\sigma_j}(a)\mathbf{Normal}(\mu_a;\mu_a^{IRF},\nu_a^{IRF}),\end{aligned} \tag{S52}$$

$$\begin{aligned}(\mathbf{D}_n^\sigma)^{laser}_{s_n\to\sigma_j}(\mu_d,\mu_a) =& (1-p_{bd})p_{ba}\mathbf{Normal}(\mu_a;\mu_a^{IRF},\nu_a^{IRF})(\mathbf{D}_n^\sigma)^{IRF}_{s_n\to\sigma_j}(\mu_d,\emptyset)\\ &+ p_{bd}\mathbf{Normal}(\mu_d;\mu_d^{IRF},\nu_d^{IRF})(1-p_{ba})(\mathbf{D}_n^\sigma)^{IRF}_{s_n\to\sigma_j}(\emptyset,\mu_a)\\ &+ p_{bd}p_{ba}\mathbf{Normal}(\mu_d;\mu_d^{IRF},\nu_d^{IRF})\mathbf{Normal}(\mu_a;\mu_a^{IRF},\nu_a^{IRF})\\ &\times((\hat{\mathbf{D}}_n^\sigma)_{s_n\to\sigma_j}(d)+(\hat{\mathbf{D}}_n^\sigma)_{s_n\to\sigma_j}(a)).\end{aligned} \tag{S53}$$

S2.4.2 Uniform background

Finally, we incorporate uniform background, which represents the combination of all ambient light sources that emit photons with a constant rate, independent of the laser pulses. We introduce uniform background after the IRF as the arrival time distribution of these photons is not affected by the IRF, remaining uniform over the entire interpulse window. Once again, using the form for a generic light source from Eq. S38, we describe uniform background as

$$p_{dbg}(w_n) = \begin{cases}(1-p_{dd})(1-p_{da}) & w_n=(\emptyset,\emptyset)\\ p_{dd}(1/\tau)(1-p_{da}) & w_n=(\mu_d,\emptyset)\\ (1-p_{dd})p_{da}(1/\tau) & w_n=(\emptyset,\mu_a)\\ p_{dd}p_{da}(1/\tau)^2 & w_n=(\mu_d,\mu_a)\end{cases}. \tag{S54}$$

Combining this source with $(\mathbf{D}_n^\sigma)^{laser}_{s_n\to\sigma_j}$ from the previous section in the same way as described in Eq. S39, we arrive at our final expression for the detection matrices as

$$(\mathbf{D}_n^\sigma)_{s_n\to\sigma_j}(w_n) = \begin{cases} p_{dbg}(\emptyset,\emptyset)(\mathbf{D}_n^\sigma)^{laser}_{s_n\to\sigma_j}(\emptyset,\emptyset) & w_n=(\emptyset,\emptyset)\\ p_{dbg}(\emptyset,\emptyset)(\mathbf{D}_n^\sigma)^{laser}_{s_n\to\sigma_j}(\mu_d,\emptyset)+p_{dbg}(\mu_d,\emptyset)(\mathbf{D}_n^\sigma)^{laser}_{s_n\to\sigma_j}(\emptyset,\emptyset)+m_d^u(\mu_d,\emptyset) & w_n=(\mu_d,\emptyset)\\ p_{dbg}(\emptyset,\emptyset)(\mathbf{D}_n^\sigma)^{laser}_{s_n\to\sigma_j}(\emptyset,\mu_a)+p_{dbg}(\emptyset,\mu_a)(\mathbf{D}_n^\sigma)^{laser}_{s_n\to\sigma_j}(\emptyset,\emptyset)+m_a^u(\emptyset,\mu_a) & w_n=(\emptyset,\mu_a)\\ p_{dbg}(\emptyset,\mu_a)(\mathbf{D}_n^\sigma)^{laser}_{s_n\to\sigma_j}(\mu_d,\emptyset)+p_{dbg}(\mu_d,\emptyset)(\mathbf{D}_n^\sigma)^{laser}_{s_n\to\sigma_j}(\emptyset,\mu_a) & w_n=(\mu_d,\mu_a)\\ +p_{dbg}(\mu_d,\mu_a)(\mathbf{D}_n^\sigma)^{laser}_{s_n\to\sigma_j}(\emptyset,\emptyset)+M_d^u(\mu_d,\mu_a)+M_a^u(\mu_d,\mu_a). & \end{cases} \tag{S55}$$

Unlike for the laser background, the terms m_d^u, m_a^u, M_d^u, and M_a^u are no longer simple to compute. Therefore, here, we present their approximate form

$$m_d^u(t, \emptyset) \approx p_{dd}(1 - p_{da}) \left[\frac{1}{\tau} (\hat{\mathbf{D}}_n^\sigma)_{s_n \to \sigma_j}^{laser}(d) - \frac{1}{\tau} \left(\int_0^t (\mathbf{D}_n^\sigma)_{s_n \to \sigma_j}^{laser}(s, \emptyset) ds \right) \right.$$
$$\left. + \left(1 - \frac{t}{\tau}\right) (\mathbf{D}_n^\sigma)_{s_n \to \sigma_j}^{laser}(t, \emptyset) \right], \tag{S56}$$

$$m_a^u(\emptyset, t) \approx (1 - p_{dd}) p_{da} \left[\frac{1}{\tau} (\hat{\mathbf{D}}_n^\sigma)_{s_n \to \sigma_j}^{laser}(a) - \frac{1}{\tau} \left(\int_0^t (\mathbf{D}_n^\sigma)_{s_n \to \sigma_j}^{laser}(s, \emptyset) ds \right) \right.$$
$$\left. + \left(1 - \frac{t}{\tau}\right) (\mathbf{D}_n^\sigma)_{s_n \to \sigma_j}^{laser}(t, \emptyset) \right], \tag{S57}$$

$$M_d^u(t_d, t_a) \approx p_{dd} p_{da} \left[\frac{1}{\tau} (\hat{\mathbf{D}}_n^\sigma)_{s_n \to \sigma_j}^{laser}(d) - \frac{1}{\tau} \left(\int_0^{t_d} (\mathbf{D}_n^\sigma)_{s_n \to \sigma_j}^{laser}(s, \emptyset) ds \right) \right.$$
$$\left. + \left(1 - \frac{t}{\tau}\right) (\mathbf{D}_n^\sigma)_{s_n \to \sigma_j}^{laser}(t_d, \emptyset) \right] \frac{1}{\tau}, \tag{S58}$$

$$M_a^u(t_d, t_a) \approx p_{dd} p_{da} \frac{1}{\tau} \left[\frac{1}{\tau} (\hat{\mathbf{D}}_n^\sigma)_{s_n \to \sigma_j}^{laser}(a) - \frac{1}{\tau} \left(\int_0^{t_a} (\mathbf{D}_n^\sigma)_{s_n \to \sigma_j}^{laser}(s, \emptyset) ds \right) \right.$$
$$\left. + \left(1 - \frac{t}{\tau}\right) (\mathbf{D}_n^\sigma)_{s_n \to \sigma_j}^{laser}(t_a, \emptyset) \right]. \tag{S59}$$

Here, we derived the most general likelihood for smFRET under pulsed illumination and used it in our analysis. However, as mentioned earlier, this likelihood can be much simplified to an approximate form by ignoring the terms associated to the competitions between photons reaching to the detectors in $(\mathbf{D}_n^\sigma)_{s_n \to \sigma_j}^{laser}$ (Eqs. S50-S53) and $(\mathbf{D}_n^\sigma)_{s_n \to \sigma_j}$ (Eq. S59).

S3 Model Structure and Priors

After deriving the likelihood, in this section, we present our priors to derive the parametric and nonparametric posteriors. The parameters are arranged according to their hierarchical dependency, meaning that if a parameter depends on another, it will necessarily come after it. Parameters whose priors' parameters are set by hand rather than by another parameter are highlighted with a $(*)$.

S3.1 Parametric Model

$$
\begin{aligned}
\boldsymbol{\pi}_0 &\sim \textbf{Dirichlet}(1, 1, \ldots, 1), && (*) \\
\boldsymbol{\pi}_m &\sim \textbf{Dirichlet}(1, 1, \ldots, 1), && m \in \{1, \ldots, M\}, \ (*) \\
s_1 &\sim \textbf{Categorical}(\pi_0), && \\
s_n | s_{n-1} &\sim \textbf{Categorical}(\pi_{s_{n-1}}), && n \in \{2, 3, \ldots, N\}, \\
\pi_{ex} &\sim \textbf{Beta}(1, 1), && (*) \\
a_n &\sim \textbf{Categorical}(1 - \pi_{ex} - k_a \pi_{ex},\ \pi_{ex},\ k_a \pi_{ex}), && n \in \{1, \ldots, N\}, \\
\lambda_d &\sim \textbf{Gamma}(1, 1), && (*) \\
\lambda_a &\sim \textbf{Gamma}(1, 1), && (*) \\
\lambda_{\sigma_m}^{FRET} &\sim \textbf{Gamma}(1, 1), && m \in \{1, \ldots, M\}, \ (*) \\
w_n &\sim p(w | a_n, \lambda_d, \lambda_a, \lambda_{s_n}^{FRET}), && n \in \{1, \ldots, N\},
\end{aligned}
$$

where the distribution $p(w|a_n, \lambda_d, \lambda_a, \lambda_{s_n}^{FRET})$ is the likelihood derived in Section S2 with $\boldsymbol{\rho}_{pulse}$ set by the auxiliary parameter a_n.

S3.2 Nonparametric Model

$$
\begin{aligned}
\gamma &\sim \textbf{Gamma}(1,1), && (*)\\
\beta &\sim \textbf{Dirichlet}\left(\frac{\gamma}{M},\ldots,\frac{\gamma}{M}\right), &&\\
\alpha &\sim \textbf{Gamma}(1,1), && (*)\\
\kappa &\sim \textbf{Beta}(\phi,1), && (*)\\
\boldsymbol{\pi}_0 &\sim \textbf{Dirichlet}(\alpha\boldsymbol{\beta}), &&\\
\boldsymbol{d}_m &= \begin{cases}(d_m)_i = 1 & i = m\\ (d_m)_i = 0 & i \neq m\end{cases}, &&\\
\boldsymbol{\pi}_m &\sim \textbf{Dirichlet}(\alpha((1-\kappa)\boldsymbol{\beta} + \kappa\boldsymbol{d}_m)), && m \in \{1,\ldots,M\},\\
s_1 &\sim \textbf{Categorical}(\boldsymbol{\pi}_0), &&\\
s_n|s_{n-1} &\sim \textbf{Categorical}(\boldsymbol{\pi}_{s_{n-1}}), && n \in \{2,3,\ldots,N\},\\
\pi_{ex} &\sim \textbf{Beta}(1,1), && (*)\\
a_n &\sim \textbf{Categorical}(1-\pi_{ex}-k_a\pi_{ex},\ \pi_{ex},\ k_a\pi_{ex}), && n \in \{1,\ldots,N\},\\
\lambda_d &\sim \textbf{Gamma}(1,1), && (*)\\
\lambda_a &\sim \textbf{Gamma}(1,1), && (*)\\
\lambda_{\sigma_m}^{FRET} &\sim \textbf{Gamma}(1,1), && m \in \{1,\ldots,M\},\ (*)\\
w_n &\sim p(w|a_n,\lambda_d,\lambda_a,\lambda_{s_n}^{FRET}), && n \in \{1,\ldots,N\},
\end{aligned}
$$

where the distribution $p(w|a_n,\lambda_d,\lambda_a,\lambda_{s_n}^{FRET})$ is the likelihood derived in Section S2 with $\boldsymbol{\rho}_{pulse}$ set by the auxiliary parameter a_n.

S4 Sampling from the Posterior: Gibbs Algorithm Steps

The central object of interest in the Bayesian paradigm is the posterior

$$p(\vartheta|w_{1:N}) \propto L(w_{1:N}|\vartheta)p(\vartheta). \tag{S60}$$

where ϑ denotes the set of all unknowns including $\boldsymbol{\rho}_{start}$, rates in $\mathbf{G}_\psi$, and transition probabilities in $\mathbf{\Pi}_\sigma$. Furthermore, $p(\vartheta)$ denotes the set of priors given in Sec. S3.

In order to infer the unknown parameters, we draw numerical samples from the posterior. One way of doing this is through Markov Chain Monte Carlo (MCMC) methods, where samples from the posterior are drawn iteratively to construct a Markov chain. In this implementation, we utilize the Gibbs algorithm, where individual parameters x are sampled from their conditional posterior distributions in each MCMC iteration

$$p(x|\vartheta/\{x\}, w_{1:N}), \tag{S61}$$

where x is some model parameter and $\vartheta/\{x\}$ represents the set of all model parameters without x. In the following, we present our Gibbs sampling steps for each parameter.

S4.1 Photophysical rates

A photophysical rate, λ, is sampled from the conditional posterior

$$p(\lambda|\vartheta/\{\lambda\}, w_{1:N}) \propto L(w_{1:N}|\vartheta)p(\lambda), \tag{S62}$$

where prior $p(\lambda)$ is the same for all the photophysical rates

$$p(\lambda) = \mathbf{Gamma}(\lambda; 1, 1). \tag{S63}$$

This particular conditional posterior does not have a closed form, so the photophysical rates are sampled through a Metropolis-Hasting (MH) procedure. We do so by proposing new values for rates as follows

$$\lambda^* \sim \mathbf{Gamma}\left(\phi, \frac{\lambda}{\phi}\right), \tag{S64}$$

where ϕ is a parameter tuned to improve mixing. Subsequently, the proposal is accepted with probability given by

$$\boldsymbol{\alpha} = \min\left\{1, \frac{L(w_{1:N}|\lambda^*)\mathbf{Gamma}(\lambda^*; 1, 1)\mathbf{Gamma}(\lambda; \phi, \frac{\lambda^*}{\phi})}{L(w_{1:N}|\lambda)\mathbf{Gamma}(\lambda; 1, 1)\mathbf{Gamma}(\lambda^*; \phi, \frac{\lambda}{\phi})}\right\}, \tag{S65}$$

where $L(w_n|\lambda)$ is the likelihood for individual pulse derived in Sec. S2.

S4.2 Excitation Probability

To allow for direct sampling of the excitation probabilities and simplify the pulse likelihood functions derived in Sec S2, we sample the photophysical state a_n immediately after the pulse. By doing so, we effectively set ρ_{pulse} to be a certain photophysical state.

Since the photophysics of the individual pulses are assumed independent, we can sample each of the a_n individually from their conditional posterior

$$p(a_n|\vartheta/\{a_n\}, w_{1:N}) \propto L_n(w_n|\vartheta)p(a_n) = L_n(w_n|\vartheta)\mathbf{Categorical}(a_n; \boldsymbol{\rho}_{pulse}), \tag{S66}$$

where, as derived in Sec. S2.1,

$$\boldsymbol{\rho}_{pulse} = \left(1 - \pi_{ex} - k_a\pi_{ex}, \quad \pi_{ex}, \quad k_a\pi_{ex}\right). \tag{S67}$$

Since a_n represents the photophysical state immediately after the pulse, it has three photophysical states ψ_1, ψ_2, and ψ_3 that represent both donor and acceptor being in the ground state, the donor being excited and the acceptor being in the ground state, and the donor being in the ground state and acceptor being excited, respectively. Therefore, we sample

$$a_n \sim \mathbf{Categorical}\left(\frac{L_n(w_n|a_n=\psi_1)\xi_1}{\sum_{i=1}^3 L_n(w_n|a_n=\psi_i)\xi_i}, \frac{L_n(w_n|a_n=\psi_2)\xi_2}{\sum_{i=1}^3 L_n(w_n|a_n=\psi_i)\xi_i}, \frac{L_n(w_n|a_n=\psi_3)\xi_3}{\sum_{i=1}^3 L_n(w_n|a_n=\psi_i)\xi_i}\right). \tag{S68}$$

Now, the excitation probability π_{ex} is sampled from the conditional posterior

$$p(\pi_{ex}|\vartheta/\{\pi_{ex}\}, w_{1:N}) \propto L(w_{1:N}|\vartheta)p(\pi_{ex}) = L(w_{1:N}|\vartheta)\mathbf{Beta}(\pi_{ex}; 1, 1), \tag{S69}$$

which has likelihood-prior conjugacy because of our choice to also sample the photophysical trajectory $a_{1:N}$. Intuitively, the number of times that the photophysical trajectory records the donor being excited is the number of "successes" of a Bernoulli random variable. Therefore, we can directly sample π_{ex} from the following probability density

$$\pi_{ex} \sim \mathbf{Beta}(1 + \sum_{i=1}^{N} \mathbb{1}\{a_i = \psi_2\}, 1 + N - \sum_{i=1}^{N} \mathbb{1}\{a_i = \psi_2\}), \tag{S70}$$

where $\sum_{i=1}^{N} \mathbb{1}\{a_i = \psi_2\}$ is the number of times that that the photophysical trajectory is in ψ_2.

S4.3 System State Trajectory

Sampling of the system state trajectory is done through a standard forward filtering backward sampling algorithm, which we briefly describe here [4]. First, we sample an initial probability vector $\boldsymbol{\pi}_0$ that is informed by the prior and the first system state of the previous trajectory.

$$\boldsymbol{\pi}_0 \sim \mathbf{Dirichlet}(\alpha((1-\kappa)\boldsymbol{\beta} + \kappa\mathbf{d}_m + \mathbf{n}_{0\cdot}), \tag{S71}$$

where $\alpha((1-\kappa)\boldsymbol{\beta} + \kappa\mathbf{d}_m$ is the prior of the transition probabilities modified with the sticky hyperparameter and $\mathbf{n}_m$ is a vector with value one at the index corresponding to s_1 and zero otherwise.

Next, we construct a forward filter by propagating forward using the transition matrix while taking into account observations. The first time level of the forward filter is given by,

$$\mathcal{A}_{1i} = \boldsymbol{\pi}_{0i} \times L_1(w_1|s_1 = \sigma_i), \qquad i = 1, \ldots, M_\sigma. \tag{S72}$$

This allows us to then move forward by computing for each n from one to N,

$$\mathcal{A}_{ni} = L_n(w_n|s_1 = \sigma_i) \sum_{i=1}^{M} \boldsymbol{\pi}_{im} \mathcal{A}_{n-1,i}, \qquad i = 1, \ldots, M_\sigma,\ n = 1, \ldots, N. \tag{S73}$$

Finally, we sample the transition by recursively sampling the system state starting at the end and moving towards the first pulse in the following way

$$s_N \sim \textbf{Categorical}(\mathcal{A}_N), \tag{S74}$$

$$s_n|s_{n+1} \sim \textbf{Categorical}(\mathbf{b}_n), \tag{S75}$$

where

$$\mathbf{b}_{ni} = \frac{\boldsymbol{\pi}_{i,s_{n+1}} \mathcal{A}_{n+1,i}}{\sum_{j=1}^{M} \boldsymbol{\pi}_{j,s_{n+1}} \mathcal{A}_{n+1,j}} \qquad , i = 1, \ldots, M_\sigma,\ n = 1, \ldots, N. \tag{S76}$$

S4.4 Transition probabilities

The transition probabilities are sampled as vectors $\boldsymbol{\pi}_m$ that represent transition probabilities out of state m from the conditional posterior

$$p(\boldsymbol{\pi}_m|\vartheta/\{\boldsymbol{\pi}_m\}, w_{1:N}) \propto L(w_{1:N}|\vartheta)p(\boldsymbol{\pi}_m) \tag{S77}$$

$$= L(w_{1:N}|\vartheta)\textbf{Dirichlet}(\boldsymbol{\pi}_m; \alpha((1-\kappa)\boldsymbol{\beta} + \kappa\mathbf{d}_m)), \tag{S78}$$

where $\alpha((1-\kappa)\boldsymbol{\beta} + \kappa\mathbf{d}_m)$ is the prior of the transition probabilities modified with the sticky hyperparameter.

These transition probability vectors are updated through likelihood-prior conjugacy with the state trajectory. Using the closed form of the conditional posterior, we sample each $\boldsymbol{\pi}_m$ through

$$\boldsymbol{\pi}_m \sim \textbf{Dirichlet}(\alpha((1-\kappa)\boldsymbol{\beta} + \kappa\mathbf{d}_m) + \mathbf{n}_{m\cdot}), \tag{S79}$$

where $\mathbf{n}_{m\cdot}$ is a vector which collects the number of each transition out of system state σ_m.

S4.5 Base Distribution

The sampling of the base distribution and the other hyperparameters are heavily inspired by the work of Emily Fox et. al. [5], and it is highly recommended for those interested in a more in-depth discussion of sticky iHMMs to read their work.

To sample the base distribution, we first sample auxiliary parameters $\mathbf{D}$ and $\mathbf{W}$. We start with $\mathbf{D}$. For each $i, j = 1, \ldots, M$, we set

$$M_{ij} = \sum_{k=0}^{\mathbf{n}_{ij}-1} \textbf{Bernoulli}\left(\frac{\alpha\beta_i}{j + \alpha\beta_i}\right). \tag{S80}$$

Next we sample $\mathbf{W}$, which intuitively represents the number of times a self-transition occurred because of the influence of the sticky hyperparameter.

$$W_{ii} = \textbf{Binomial}\left(M_{ii}, \frac{\kappa}{\kappa + \beta_i(1-\kappa)}\right). \tag{S81}$$

Finally, define $\overline{\mathbf{D}} = \mathbf{D} - \mathbf{W}$. We can now directly sample the base distribution through

$$\boldsymbol{\beta} \sim \textbf{Dirichlet}(\gamma\zeta + \sum_{i=1}^{M} \overline{\mathbf{D}}_{ij}), \tag{S82}$$

where ζ is an M dimensional vector with all elements set to $\frac{1}{M}$.

S4.6 Optional Hyperparameters

Many of these sampling steps draw on auxiliary parameters $\mathbf{D}$, $\mathbf{W}$, and $\overline{\mathbf{D}}$ described in the previous section for sampling the base distribution.

S4.6.1 Transition probabilities concentration hyperparameter

To sample the concentration parameter α, we sample additional auxiliary parameters, $\mathbf{r}$ and $\mathbf{s}$, which are vectors of size M. Define $n_{\cdot j} = \sum_{i=1}^{M} \mathbf{n}_{ij}$ as the total number of transitions into system state σ_j. We then sample

$$\mathbf{r}_i \sim \textbf{Beta}(1+\alpha, n_j), \tag{S83}$$

$$\mathbf{s}_i \sim \textbf{Bernoulli}\left(\frac{n_{\cdot j}}{n_{\cdot j} + \alpha}\right). \tag{S84}$$

We can then sample

$$\alpha \sim \textbf{Gamma}(\alpha + \sum_{i=1}^{M}\sum_{j=1}^{M} \mathbf{D}_{ij} - \sum_{i=1}^{M} \mathbf{s}, 1 - \sum_{i=1}^{M} \log(r_i)). \tag{S85}$$

S4.6.2 Sticky hyperparameter

Naturally the sticky hyperparameter is updated by taking the number of times self transitions occur due to the stickiness (found in $\mathbf{W}$) and using those as successes to update a Beta distribution. The sampling step is given as

$$\kappa \sim \textbf{Beta}\left(1 + \sum_{i=1}^{M} \mathbf{W}_{ii}, \phi + \sum_{i=1}^{M}\sum_{j=1}^{M} \mathbf{D}_{ij} - \sum_{i=1}^{M} \mathbf{W}_{ii}\right), \tag{S86}$$

where ϕ is a preset parameter that controls the "stickiness" of the HMM.

S4.6.3 Base distribution concentration hyperparameter

The base distribution concentrion parameter γ requires sampling additional parameters c and p. Additionally, define K as the number of elements of $\overline{\mathbf{D}}$ that are greater than zero. Next, we sample

$$c \sim \mathbf{Beta}\left(\gamma + 1, \sum_{i=1}^{M}\sum_{j=1}^{M}\overline{\mathbf{D}}_{ij}\right), \tag{S87}$$

$$p \sim \mathbf{Bernoulli}\left(\frac{K}{(\sum_{i=1}^{M}\sum_{j=1}^{M}\overline{\mathbf{D}}_{ij})(1-\log(c))}\right). \tag{S88}$$

If $p = 1$, we sample γ as

$$\gamma \sim \mathbf{Gamma}(1 + K, 1 - \log(c)), \tag{S89}$$

and otherwise we sample

$$\gamma \sim \mathbf{Gamma}(K, 1 - \log(c)). \tag{S90}$$

S5 Estimation of pre-set Parameters

Parameters associated with photon detection such as crosstalk, IRF, detection efficiency, and direct acceptor excitation are preset according to the experimental conditions. Both the label and detector quantum efficiencies are combined into η_d and η_a. We preprocess only two parameters: IRF, by fitting IRF data to a Gaussian distribution; and background emission, which we deterimine individually.

S5.1 IRF

IRF data was obtained using water scattering, *i.e.*, shining the laser at a sample of water and recording the microtimes. The resulting distribution records the instrument response function, since the photons from water scattering do not experience delays due to lifetime. The expression for IRF fit using MATLAB's pre-built curve fitting tools.

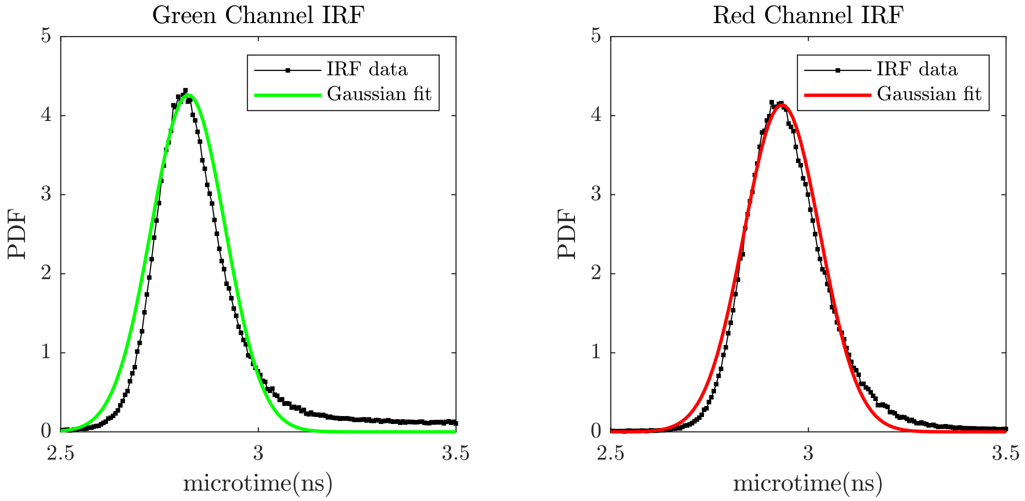

Figure S1: **IRF curve fitting.** We fit a Guassian distribution to the IRF calibration data obtained by water scattering.

S5.2 Background

The probability of receiving a background photon is considered constant over the course of the experiment. Let this probability be π_o. Consider a period of time with no emitting sample with pulses $1, \ldots, N$. Let b_n be a label for all the pulses where $b_n = 1$ if a background photon is received in one channel and $b_n = 0$ otherwise. Given that a background photon is received, it can either come from a laser source or a uniform source. Let the probability that arrives in the laser source π_b. Let c_k be a label for all background photons received with $c_k = 1$ if a photon is from the laser source, and $c_n = k$ if it is from the uniform source. In

this case, we model background by

$$b_n \sim \mathbf{Bernoulli}(\pi_o), \tag{S91}$$

$$c_k | b_n = 1 \sim \mathbf{Bernoulli}(\pi_b), \tag{S92}$$

$$t_k \sim c_k \mathbf{Normal}(\mu_{IRF}, \sigma_{IRF}^2) + (1 - c_k)\mathbf{Uniform}([0, T]). \tag{S93}$$

Since the b_n are known, we can directly obtain a maximum likelihood estimate for π_o. Let $K = \sum_{i=1}^{N} b_n$. Then the estimate is given by

$$\pi_o^* = \frac{K}{N}. \tag{S94}$$

We can then obtain an estimate for π_b through

$$\pi_b^* = \arg\max_{\pi_b} \left(\prod_{i=1}^{K} \left(\pi_b \mathbf{Normal}(t_k; \mu_{IRF}, \sigma_{IRF}^2) + (1 - \pi_b)\mathbf{Uniform}([0, T]) \right) \right). \tag{S95}$$

The laser, p_b, and dark, p_d, background probabilities are then

$$p_b = \pi_0^* \pi_b^*, \tag{S96}$$

$$p_d = \pi_0^* (1 - \pi_b^*). \tag{S97}$$

An identical calculation is done for each channel.

S6 Parameters Used for Synthetic Data Generation

Here, we detail the parameters used to produce synthetic data. Since the synthetic data algorithm incorporates crosstalk, detector efficiency, IRF, and background emissions, all of these must be set. In Table S6, the parameters not included are set according to Table S6.

Quantity	Value Assigned	Notes
$\lambda_{\sigma_1 \to \sigma_2}$	$40\,\mathrm{s}^{-1}$	Ref. [6]
$\lambda_{\sigma_2 \to \sigma_1}$	$40\,\mathrm{s}^{-1}$	Ref. [6]
$\lambda_{\sigma_1}^{FRET}$	$0.5\,\mathrm{ns}^{-1}$	from experimental data
$\lambda_{\sigma_2}^{FRET}$	$0.1\,\mathrm{ns}^{-1}$	from experimental data
π_{ex}	5×10^{-4}	Ref. [6]
λ_d	$0.35\,\mathrm{ns}^{-1}$	similar to ATTO 532 [7]
λ_a	$0.25\,\mathrm{ns}^{-1}$	similar to ATTO 647N [7]
μ_{IRF}	$2.9\,\mathrm{ns}$	from experimental data
σ_{IRF}^2	$0.001\,\mathrm{ns}^2$	from experimental data
ϕ_{da}	0.03	from experimental data
ϕ_{ad}	0.01	from experimental data
p_{bd}	$0.05\pi_{ex}$	from experimental data
p_{ba}	$0.045\pi_{ex}$	from experimental data
p_{dd}	$0.05\pi_{ex}$	from experimental data
p_{da}	$0.005\pi_{ex}$	from experimental data
η_d	0.38	experimental data and Ref. [7]
η_a	0.19	experimental data and Ref. [7]

Table S2: **Parameter values for system with two states.** Most of these values were motivated by the experimental smFRET traces gathered for this paper.

Quantity	Value Assigned	Notes
$\lambda_{\sigma_1 \to \sigma_2}$	$1200\,\mathrm{s}^{-1}$	informed by 1 mM $MgCl_2$ HJ dynamics [8]
$\lambda_{\sigma_2 \to \sigma_1}$	$1200\,\mathrm{s}^{-1}$	informed by 1 mM $MgCl_2$ HJ dynamics [8]
$\lambda_{\sigma_2 \to \sigma_3}$	$1200\,\mathrm{s}^{-1}$	informed by 1 mM $MgCl_2$ HJ dynamics [8]
$\lambda_{\sigma_3 \to \sigma_2}$	$1200\,\mathrm{s}^{-1}$	informed by 1 mM $MgCl_2$ HJ dynamics [8]
$\lambda_{\sigma_1}^{FRET}$	$0.1\,\mathrm{ns}^{-1}$	from experimental data
$\lambda_{\sigma_2}^{FRET}$	$0.4\,\mathrm{ns}^{-1}$	from experimental data
$\lambda_{\sigma_3}^{FRET}$	$0.8\,\mathrm{ns}^{-1}$	from experimental data
π_{ex}	7.5×10^{-3}	highest value obtained from experimental data

Table S3: **Parameter values for system with three system states** Values that are not specified here are identical to those in Table S6 since they are set by the experimental setup and do not change from time trace to time trace.

S7 Additional Parameter Estimates

The following figures depict the posterior distributions over all the parameters not presented in the main text.

S7.1 Synthetic Data with Two System States

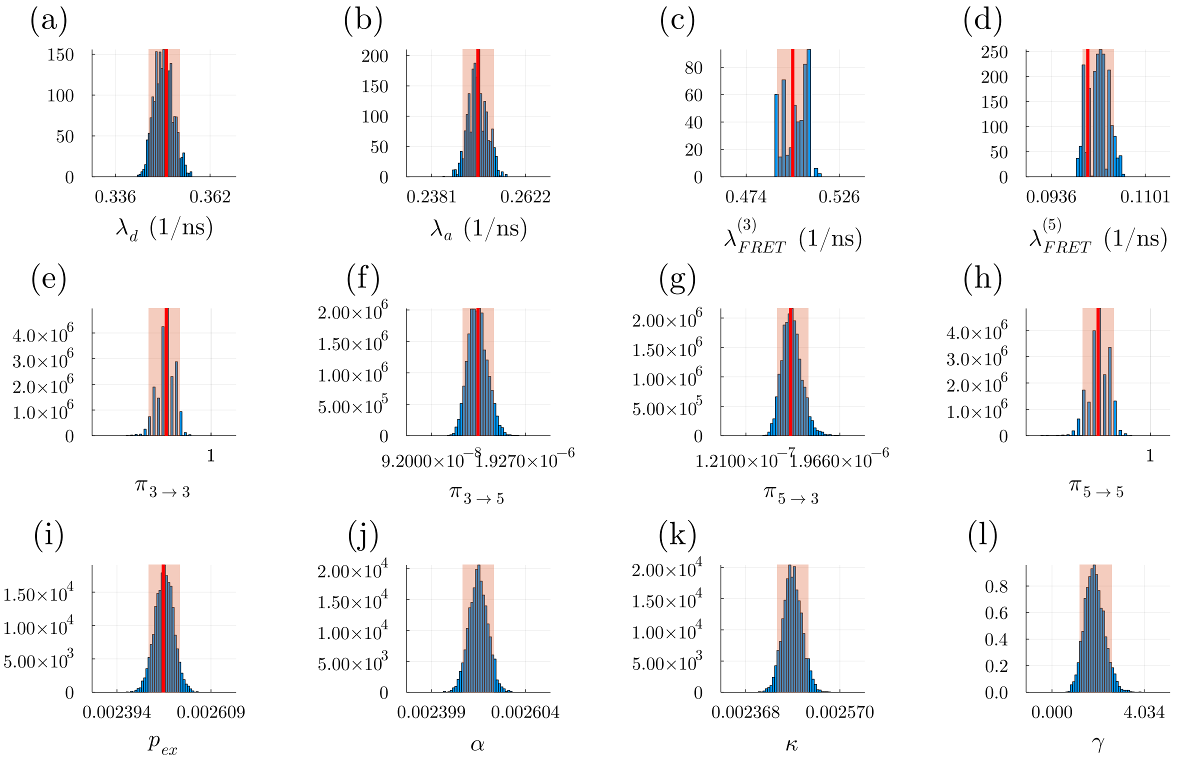

Figure S2: **Learned parameters for synthetic data with two system states.** The panels are as follows: a) donor relaxation rate; b) acceptor relaxation rate; c-d) FRET rates; e-h) system state transition probabilities for the visited states; i) excitation probability; and j-l) hyperparameters of the nonparameteric scheme, which we sample to improve mixing of the MCMC chain. The shaded regions and red lines, respectively, represent the 95% confidence interval and ground truths. The ground truth is not included for hyperparameters which are not physical quantities. The same convention is followed in the remaining figures.

S7.2 Synthetic Data with Three System States

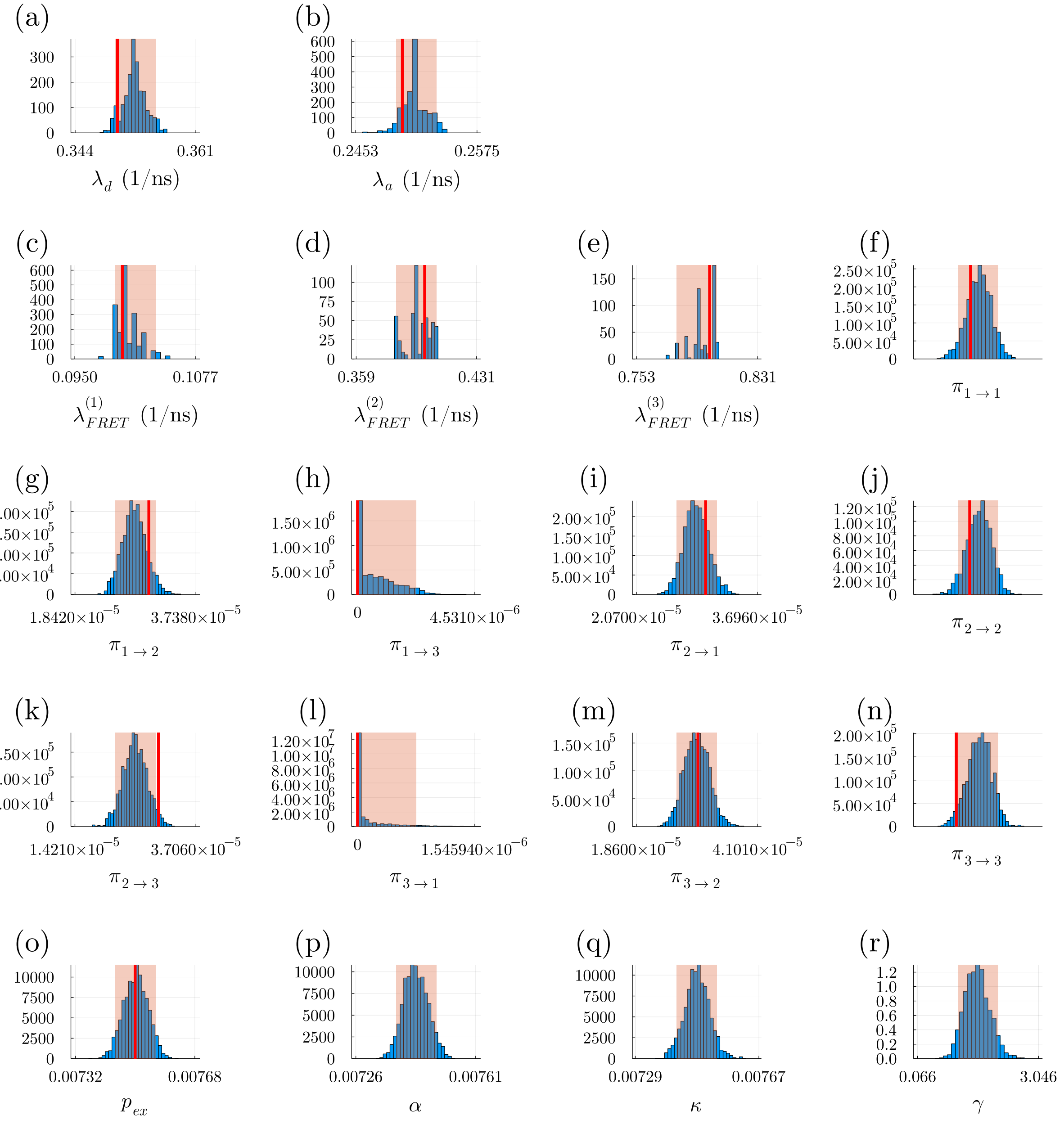

Figure S3: **Learned Parameters for Synthetic Data with three System States.** The panels are as follows: a) donor relaxation rate; b) acceptor relaxation rate; c-e) FRET rates; f-n) system state transition probabilities for the visited states; o) excitation probability; and p-r) hyperparameters of the nonparameteric scheme, which we sample for improved mixing of MCMC chain. The figure conventions are the same as those in Fig. S7.1.

S7.3 Experimental Data: 1 mm

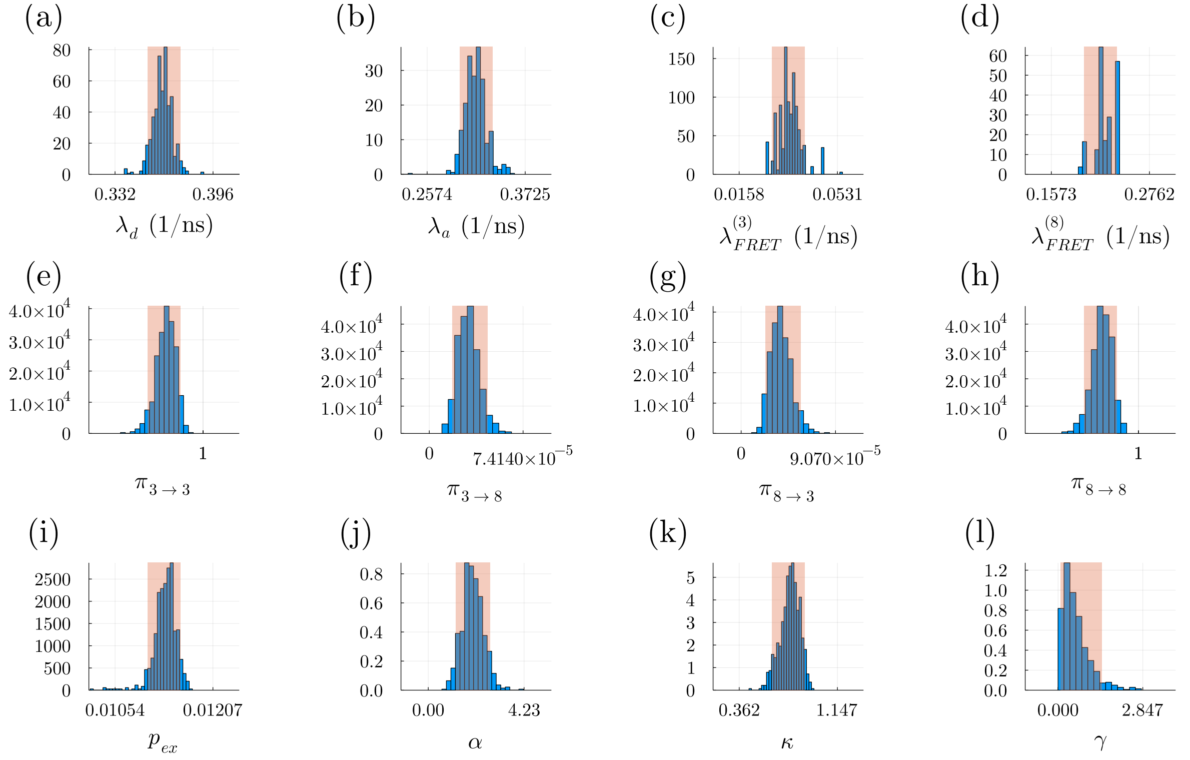

Figure S4: **Learned Parameters for 1 mm $MgCl_2$ Experimental data.** The panels are as follows: a) donor relaxation rate; b) acceptor relaxation rate; c-d) FRET rates; e-h) system state transition probabilities; i) excitation probability; and j-l) hyperparameters of the nonparameteric scheme, which we sample for improved mixing of MCMC chain. The figure conventions are the same as those in Fig. S7.1.

S7.4 Experimental Data: 3 mm

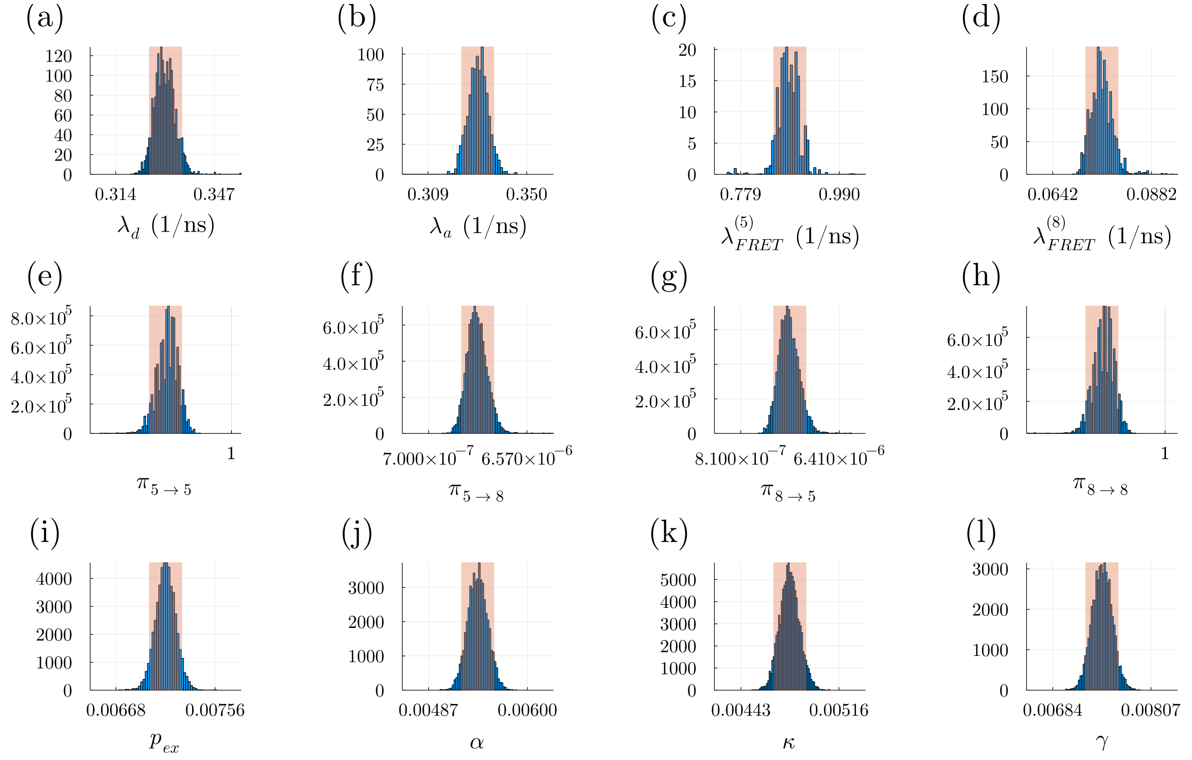

Figure S5: **Learned Parameters for 3 mm $MgCl_2$ Experimental data.** The panels are as follows: a) donor relaxation rate; b) acceptor relaxation rate; c-d) FRET rates; e)-h) system state transition probabilities for the visited states; i) excitation probability; and j-l) hyperparameters of the nonparameteric scheme, which we sample for improved mixing of MCMC chain. The figure conventions are the same as those in Fig. S7.1.

S7.5 Experimental Data: 5 mm

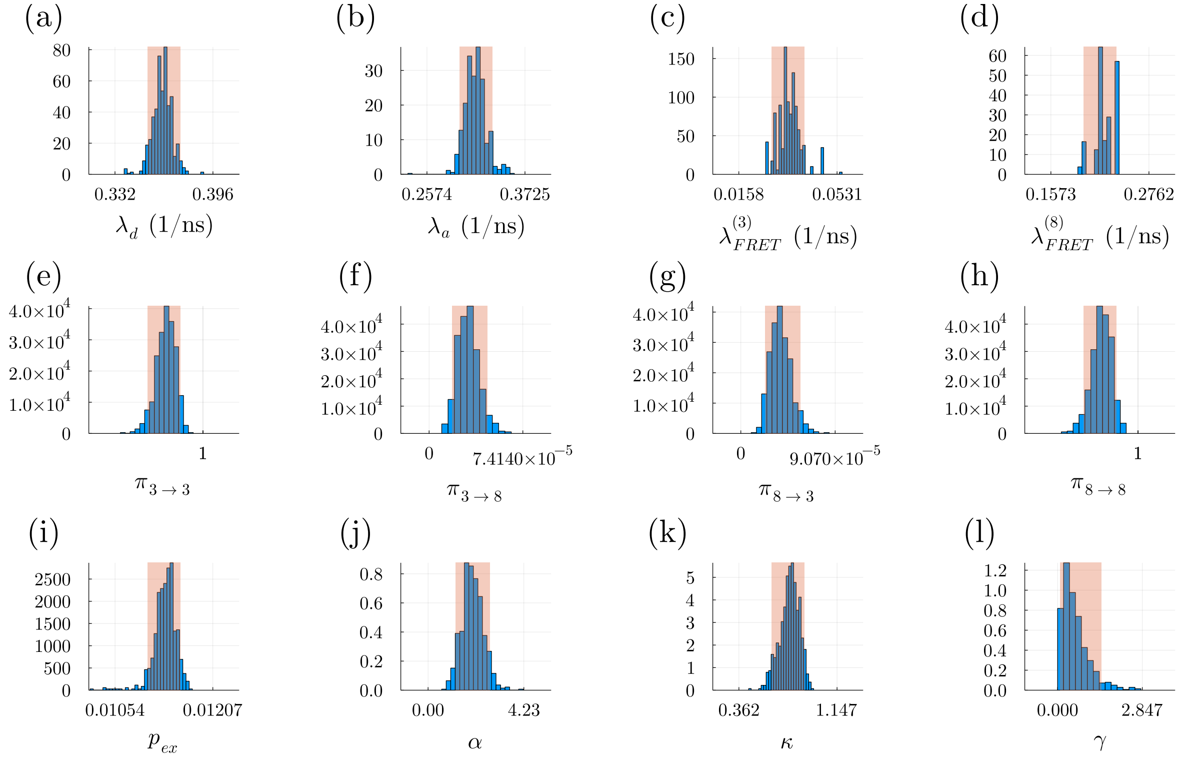

Figure S6: **Learned Parameters for 5 mm $MgCl_2$ Experimental data.** The panels are as follows: a) donor relaxation rate; b) acceptor relaxation rate; c-d) FRET rates; e-h) system state transition probabilities for the visited states; i) excitation probability; and j-l) hyperparameters of the nonparameteric scheme, which we sample for improved mixing of MCMC chain. The figure conventions are the same as those in Fig. S7.1.

S7.6 Experimental Data: 10 mm

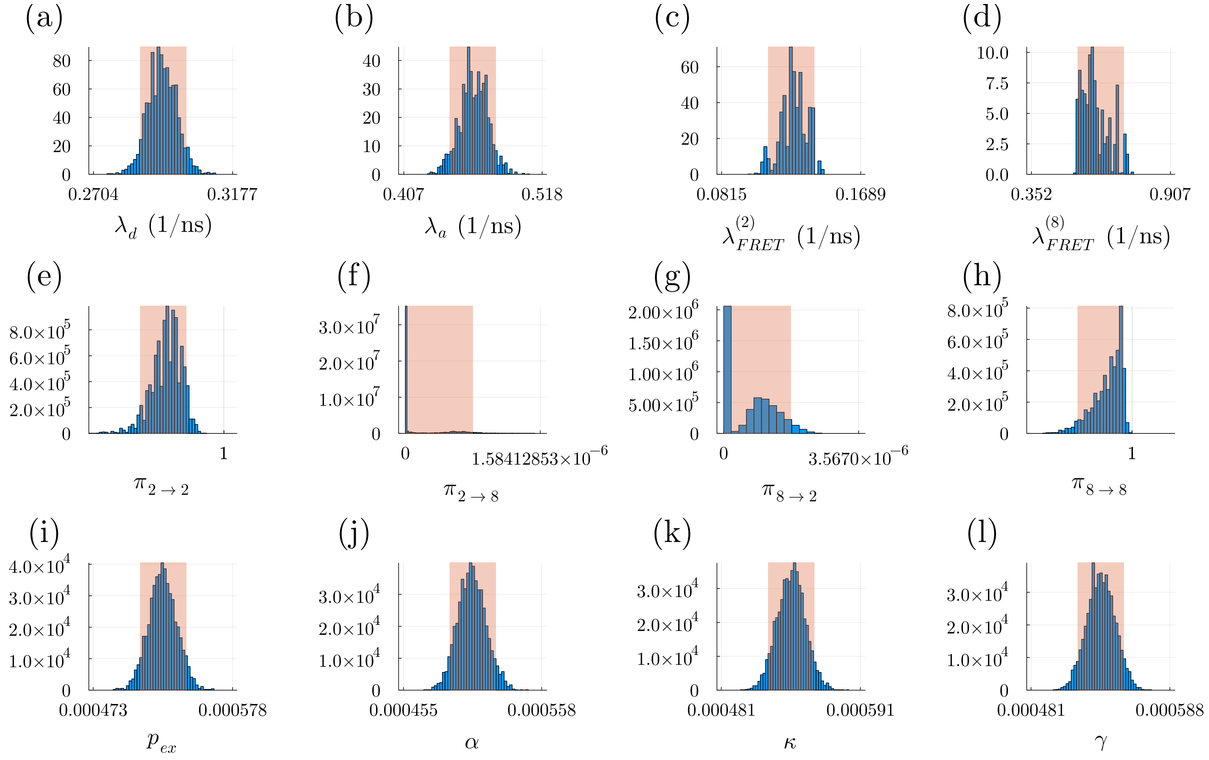

Figure S7: **Learned Parameters for 10 mm $MgCl_2$ Experimental data.** The panels are as follows: a) donor relaxation rate; b) acceptor relaxation rate; c)-d) FRET rates; e)-h) system state transition probabilities for the visited states; i) excitation probability; and j)-l) hyperparameters of the nonparameteric scheme, which we sample for improved mixing of MCMC chain. The figure conventions are the same as those in Fig. S7.1.

Bibliography

[1] Matthew Safar, Ayush Saurabh, Bidyut Sarkar, Mohamadreza Fazel, Kunihiko Ishii, Tahei Tahara, Ioannis Sgouralis, and Steve Pressé. Single photon smFRET. III. application to pulsed illumination. *bioRxiv*, 2022.

[2] Ayush Saurabh, Matthew Safar, Ioannis Sgouralis, Mohamadreza Fazel, and Steve Pressé. Single photon smFRET. I. theory and conceptual basis. *bioRxiv*, 2022.

[3] Katsumasa Fujita, Minoru Kobayashi, Shogo Kawano, Masahito Yamanaka, and Satoshi Kawata. High-resolution confocal microscopy by saturated excitation of fluorescence. *Phys. Rev. Lett.*, 99:228105, Nov 2007.

[4] L.R. Rabiner. A tutorial on hidden markov models and selected applications in speech recognition. *Proceedings of the IEEE*, 77(2):257–286, 1989.

[5] Emily B. Fox, Erik B. Sudderth, Michael I. Jordan, and Alan S. Willsky. A sticky HDP-HMM with application to speaker diarization. *The Annals of Applied Statistics*, 5(2A):1020 – 1056, 2011.

[6] Zeliha Kilic, Ioannis Sgouralis, Wooseok Heo, Kunihiko Ishii, Tahei Tahara, and Steve Pressé. Extraction of rapid kinetics from smFRET measurements using integrative detectors. *Cell Reports Physical Science*, 2(5):100409, May 2021.

[7] Atto-Tec GmbH product catalogue, 2021. Available: `https://www.atto-tec.com/produkte/Fluorescent-Labels/`, last accessed on 05/25/2022.

[8] Wooseok Heo, Kazuto Hasegawa, Kenji Okamoto, Yasushi Sako, Kunihiko Ishii, and Tahei Tahara. Scanning two-dimensional fluorescence lifetime correlation spectroscopy: Conformational dynamics of DNA Holliday junction from microsecond to subsecond. *The Journal of Physical Chemistry Letters*, 13(5):1249–1257, February 2022.
